# Constructing a ceRNA-immunoregulatory network associated with the development and prognosis of human atherosclerosis through weighted gene co-expression network analysis

**DOI:** 10.18632/aging.202486

**Published:** 2021-01-17

**Authors:** Yaozhong Liu, Na Liu, Qiming Liu

**Affiliations:** 1 Department of Cardiovascular Medicine, Second Xiangya Hospital, Central South University, Changsha, Hunan Province, China

**Keywords:** atherosclerosis, ceRNA, macrophage, ischemic event, nomogram

## Abstract

There is now overwhelming experimental and clinical evidence that atherosclerosis (AS) is a chronic inflammatory disease. The recent discovery of a new group of mediators known as competing endogenous RNA (ceRNA) offers a unique opportunity for investigating immunoregulation in AS. In this study, we used gene expression profiles from GEO database to construct a lncRNA-miRNA-mRNA ceRNA network during AS plaque development through weighted gene co-expression network analysis (WGCNA). GO annotation and pathway enrichment analysis suggested that the ceRNA network was mainly involved in the immune response. CIBERSORT and GSVA were used to calculate the immune cell infiltration score and identified macrophage as hub immunocyte in plaque development. A macrophage related ceRNA subnetwork was constructed through correlation analysis. Samples from Biobank of Karolinska Endarterectomy (BiKE) were used to identify prognostic factors from the subnetwork and yielded 7 hub factors that can predict ischemic events including macrophage GSVA score and expression value of AL138756.1, CTSB, MAFB, LYN, GRK3, and BID. A nomogram based on the key factors was established. GSEA identified that the PD1 signaling pathway was negatively associated with these prognostic factors which may explain the cardiovascular side effect of immune checkpoint therapy in anti-tumor treatment.

## INTRODUCTION

Atherosclerosis (AS) is a chronic disease characterized by lipid deposition in the vessel wall that leads to an inflammatory and proliferative cascade [1]. Myocardial infarction (MI) and stroke, the common complications of AS, represent the most common cause of death worldwide [2, 3]. Despite its epidemiological importance, the fundamental mechanisms of AS remain poorly understood and there is an urgent need to investigate the molecular pathways responsible for AS development and to identify diagnostic and prognostic biomarkers.

The immune system plays a crucial role in various stages of AS [4]. In AS, hypercholesterolemia leads to the accumulation of plasma LDL (low-density lipoprotein) in the artery wall which elicits local inflammation with an influx of monocytes that differentiate into macrophage [5]. These mononuclear phagocytes express scavenger receptors that permit them to bind lipoprotein particles and become foam cells [6]. T lymphocytes, although numerically less abundant than monocytes, also enter the intima and regulate functions of the innate immune cells as well as the endothelial cells (ECs) and smooth muscle cells (SMCs) [7, 8].

Long noncoding RNAs (lncRNAs) are a type of RNA over 200 nucleotides in length [9]. Although they do not participate in protein coding, increasing evidence has made it clear that lncRNAs can have numerous biological and molecular functions such as epigenetic regulation, signal transduction, cell differentiation, and immunoregulation [10]. Furthermore, sufficient information has demonstrated that lncRNAs can take part in the development of diverse cardiovascular diseases through interactions with microRNAs (miRNAs) or messenger RNAs (mRNAs) [11]. This competing endogenous RNA (ceRNA) hypothesis proposed by Salmena et al. has attracted increasing attention [12]. In this hypothesis, lncRNAs transcripts act as ceRNAs or natural microRNA sponges—they communicate with and co-regulate each other and mRNAs by competing for binding to shared miRNAs, a family of small non-coding RNAs that are important post-transcriptional regulators of gene expression [13]. In recent years, researchers have begun to shed light on the role of ceRNA in AS [14]. For instance, the LncRNA MIAT can sponge miR-149-5p and inhibit efferocytosis in advanced atherosclerosis through upregualting CD47 [15].

**Figure 1:**
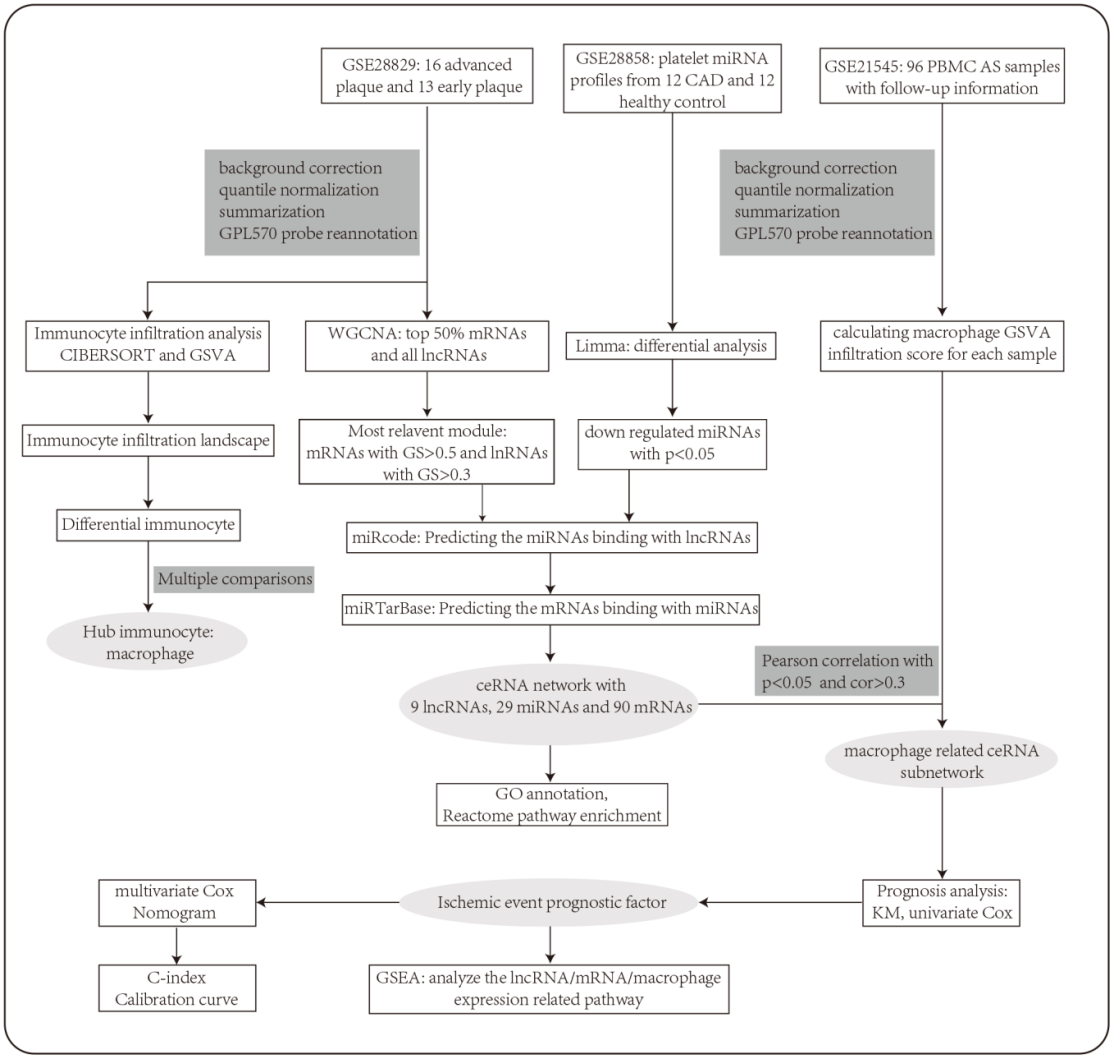
**The workflow of the integrative bioinformatics analyses.**

We should note that the detailed mechanism of immunoregulation during AS remains poorly understood. Besides, the role of ceRNA in immunoregulation during AS development has not been investigated. What’s more, no prognostic biomarkers based on a ceRNA immunoregulatory network have even been identified in AS. In this study (Figure 1), we constructed a ceRNA network in AS by using the method of weighted gene co-expression network analysis (WGCNA), which makes sure that the nodes are highly interconnected and that the network is highly reliable. We then conducted the immune cell infiltration analysis and constructed a macrophage related ceRNA subnetwork. Further, we identified several key factors in the subnetwork that can be prognostic biomarkers for ischemic events of AS. A nomogram based on these hub factors was then constructed. Findings from our study may contribute to the development of therapeutic targets and molecular prognostic tools in AS.

## RESULTS

### Construction of co-expression modules and identification of key module

**Figure 2:**
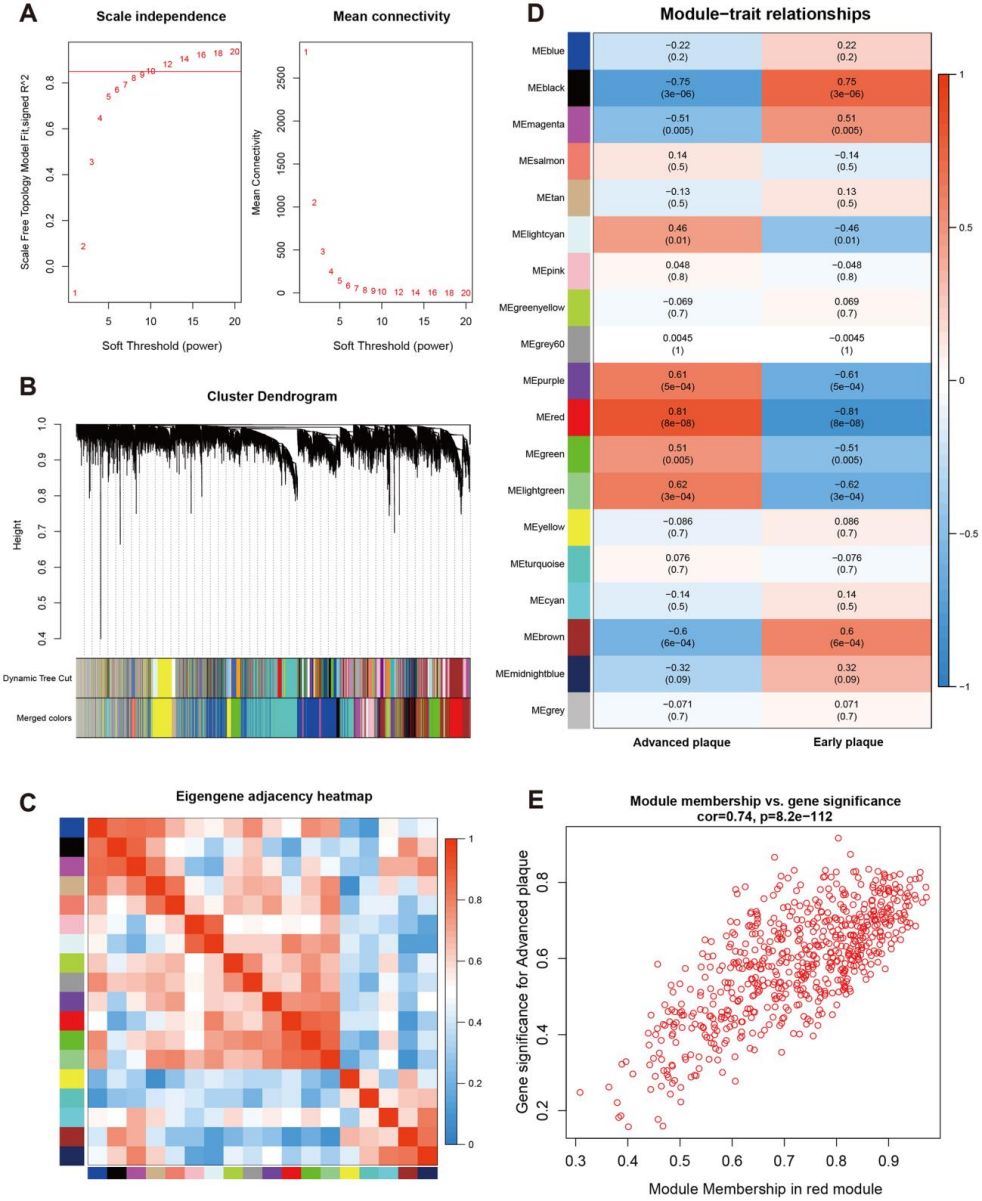
**Construction of weighted co-expression network and module analysis.** (**A**) Soft threshold selection process; (**B**) Cluster dendrogram. Each color represents one specific co-expression module. In the colored rows below the dendrogram, the two colored rows represent the original modules and merged modules; (**C**) Eigengene adjacency heatmap of different modules; (**D**) Heatmap of the correlation between status (advanced and early plaque) and module eigengenes. Each row corresponds to a module eigengene, and each column corresponds to a trait. Each cell contains the corresponding correlation (first line) and P-value (second line). The table is color-coded by correlation according to the color legend. P-value < 0.05 represents statistical significance; (**E**) Correlation between module membership of Red module and gene significance with advanced plaque (absolute value).

A β value of 10 was used to obtain the approximate scale-free topology with a scale-free topology fit index > 0.85 at the lowest power (Figure 2A). Next, dynamic tree cutting was used to produce co-expression modules and 19 modules were generated in the co-expression network (Figure 2B). The interaction and connectivity of eigengenes among different gene co-expression modules were plotted in Figure 2C. We then calculated and plotted the relation of each module with their corresponding clinical traits. From Figure 2D, we could conclude that the Red module revealed the strongest correlation (module-trait weighted correlation = 0.81, p < 0.001) with advanced atherosclerosis plaque and was identified as key module for plaque development. The significant correlation between module membership (MM) in the Red module and gene significance (GS) for an advanced plaque is presented in Figure 2E. The most significantly enriched biological process term of each module was summarized in Table 1.

**Table 1:** Gene ontology biological process enrichment analysis of modules.

**model color**	**number of mRNAs**	**most significant term**	**p.adjusted**
blue	1589	GO:0071806 protein transmembrane transport	1.15E-03
black	363	GO:0042659 regulation of cell fate specification	1.07E-01
magenta	264	GO:0048193 Golgi vesicle transport	2.88E-01
salmon	92	GO:0070125 mitochondrial translational elongation	6.95E-12
tan	73	GO:0045116 protein neddylation	2.85E-01
lightcyan	78	GO:0000380 alternative mRNA splicing, via spliceosome	1.64E-01
pink	296	GO:0010603 regulation of cytoplasmic mRNA processing body assembly	5.66E-04
greenyellow	98	GO:0050806 positive regulation of synaptic transmission	1.68E-02
grey60	67	GO:0043062 extracellular structure organization	1.75E-04
purple	146	GO:0030098 lymphocyte differentiation	4.25E-04
red	595	GO:0042119 neutrophil activation	1.62E-24
green	798	GO:0010631 epithelial cell migration	3.48E-08
lightgreen	57	GO:2001238 positive regulation of extrinsic apoptotic signaling pathway	2.84E-01
yellow	298	GO:0003214 cardiac left ventricle morphogenesis	9.64E-02
turquoise	1346	GO:0023061 signal release	1.04E-01
cyan	83	GO:0043434 response to peptide hormone	4.85E-03
brown	856	GO:0030198 extracellular matrix organization	1.26E-07
midnightblue	85	GO:0008380 RNA splicing	1.20E-04
grey	513	GO:0010721 negative regulation of cell development	3.17E-03

### CeRNA network in AS plaque development

**Figure 3:**
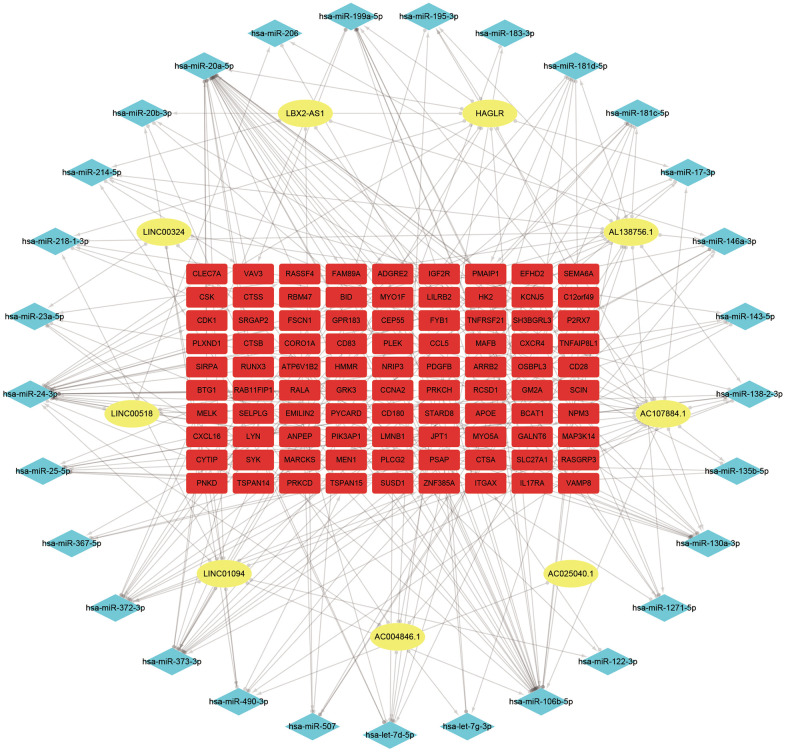
**ceRNA network of the 9 lncRNAs, 29 miRNAs, and 90 mRNAs.** Note: Blue diamond represents lncRNAs, yellow round denotes miRNAs, and red square represents mRNAs.

127 downregulated miRNAs were identified from GSE28858. They were sent for ceRNA construction along with lncRNAs/mRNAs with a GS > 0.3/0.5 in the Red module. Firstly, based on the miRcode database that matches potential miRNAs with lncRNAs, a total of 80 lncRNA-miRNA pairs containing 9 lncRNAs and 32 miRNAs were identified. Next, concerning the target gene predictions of the 32 miRNAs, we used the miRTarBase database and predicted 8308 miRNA-mRNA pairs, including 4327 target genes. Subsequently, we matched the predicted target gene with significant mRNAs in the Red module. Finally, we constructed a lncRNA-miRNA-mRNA network which includes 9 lncRNAs, 29 miRNAs, 90 mRNAs, and visualized them in Cytospace software (Figure 3). Through the above stringent screening and prediction protocol, the constructed ceRNA networks were therefore considered highly interconnected and may contribute to AS plaque development.

### Enrichment analysis and PPI analysis

**Figure 4:**
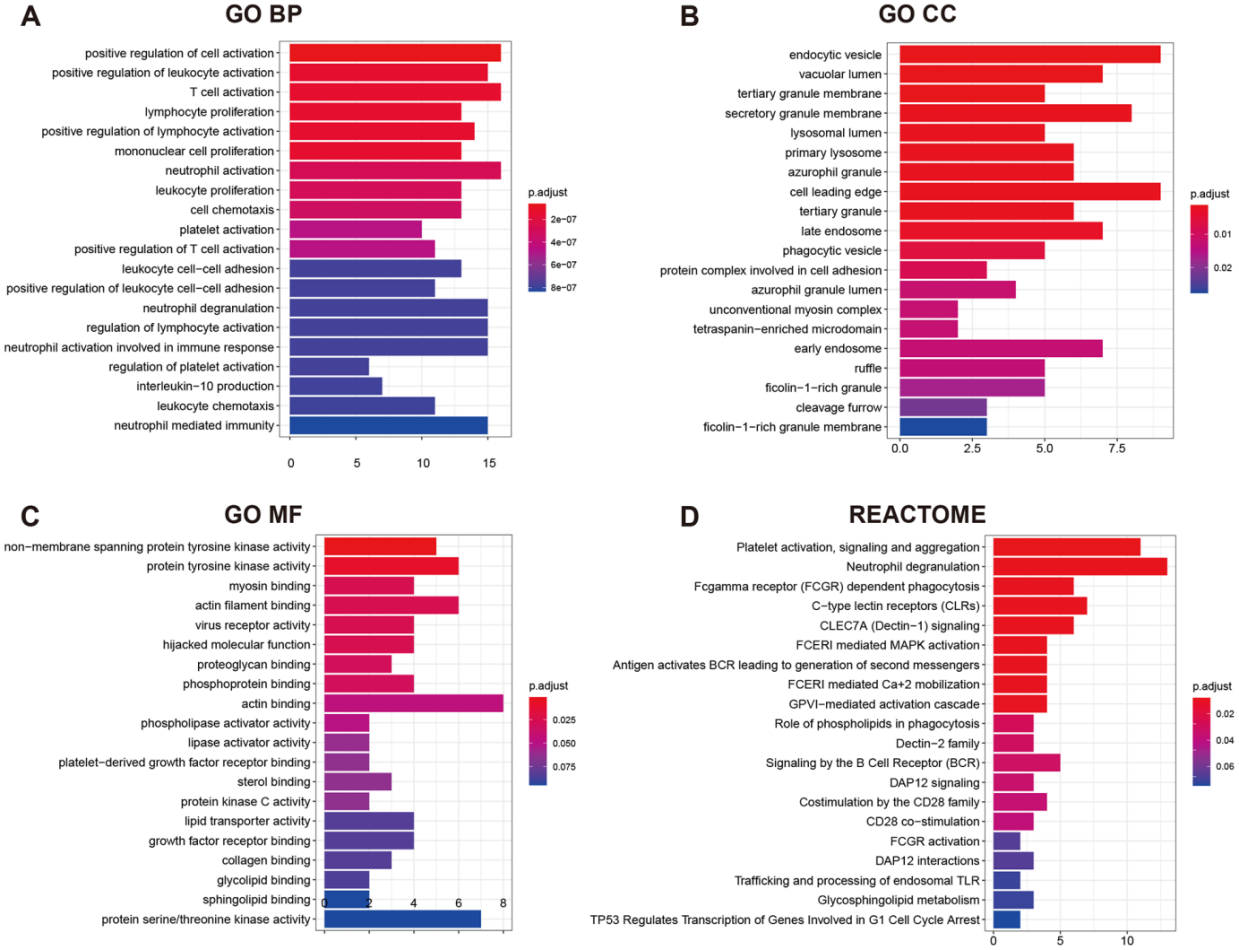
**GO functional annotation and Reactome pathway enrichment analysis for the 90 mRNAs.** (**A**) Top 20 enriched biological process; (**B**) Top 20 enriched cellular component; (**C**) Top 20 enriched molecular function; (**D**) Top 20 enriched Reactome pathway.

**Figure 5:**
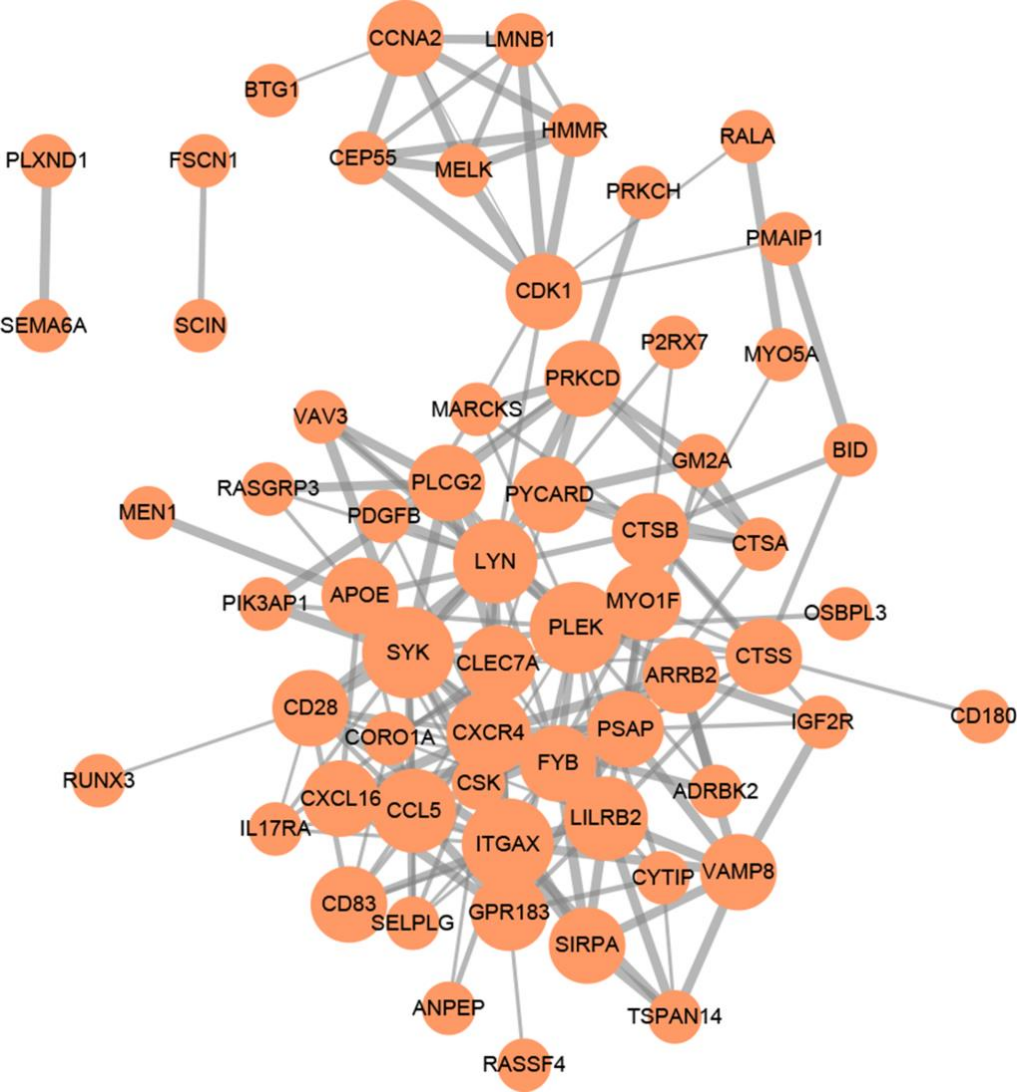
**PPI network of 90 mRNAs based on the STRING database.** Each node represents a protein-coding gene. The size of each node is mapped to its degree. Terms with a combined interaction score > 0.4 are linked by an edge (the thickness of the edge represents the interaction score).

GO (gene ontology) annotation (BP: biological process; CC: cellular component; MF: molecular function) and Reactome pathway enrichment analyses were conducted to clarify the biological functions of the ceRNA network by inputting the list of 90 target mRNAs. As presented in Figure 4A–4C, the enriched GO annotations include positive regulation of leukocyte activation, T cell activation, mononuclear cell proliferation in the BP category; endocytic vesicle, vacuolar lumen, lysosomal lumen in the CC category; and protein tyrosine kinase activity, actin filament binding, virus receptor activity in the MF category. Figure 4D shows that the ceRNA network is significantly enriched in platelet activation, neutrophil degranulation, and phagocytosis. The above results suggested that the constructed ceRNA may play crucial roles in regulating the immune response during AS development. We also constructed a PPI network of the target genes with each pair having a combined score of > 0.4 (Figure 5), based on the STRING database.

### Immunocyte subtype infiltration detection

**Figure 6:**
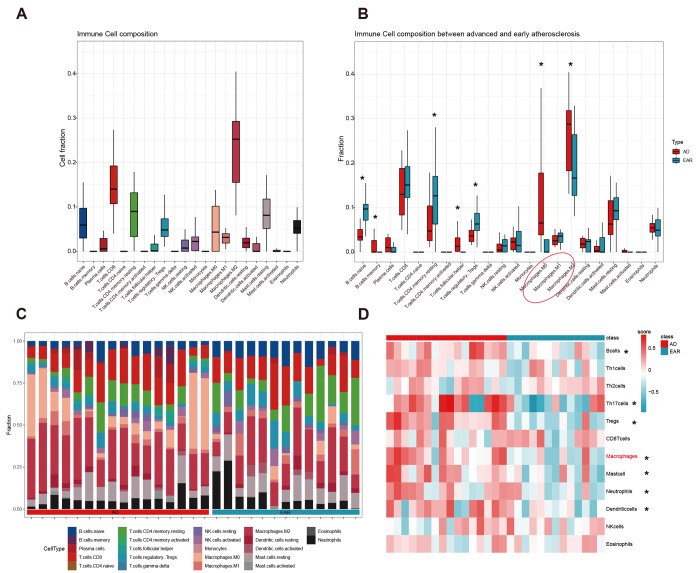
**Immune cell infiltration analysis.** (**A**) Cell composition analysis of the 29 plaque samples; (**B**) Grouped by type (advanced/early); (**C**) Scale histogram of immune cell fraction; (**D**) Heatmap of 12 immune cells GSVA score. * represents statistical significance.

We first detected the landscape of 22 immunocyte infiltration in 29 atherosclerosis plaques using the CIBERSORT algorithm. The top 3 abundant subtypes in carotid plaque were ‘Macrophage.M2’, ‘T.cells.CD8’, and ‘T.cells.CD4.memory.resting’ (Figure 6A). After the Wilcoxon rank test, 7 immunocytes were detected differentially infiltrated between the advanced and early group (Figure 6B). To make the results more reliable, we compared the infiltration score of 12 immune cells between two groups using the GSVA method (Figure 6D). By integrating the GSVA and CIBERSORT results, macrophage was then identified as hub immune cell as it was the only one to have similar trends from the two methods.

### Construction of macrophage related ceRNA subnetwork

**Figure 7:**
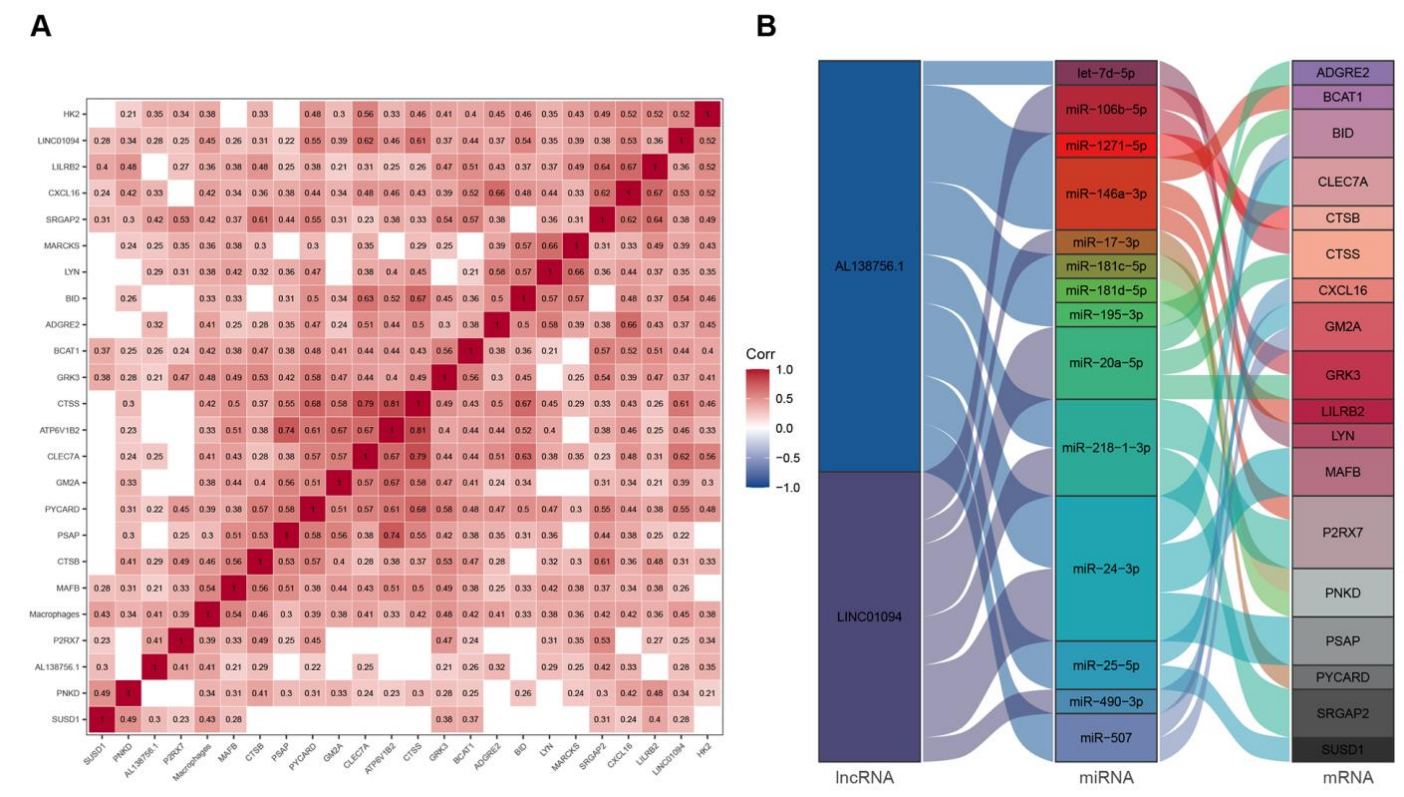
**ceRNA network associated with macrophage infiltration score.** (**A**) Pearson's correlation matrix; (**B**) Macrophage related ceRNA subnetwork. Flow indicates interaction.

We used GSE21545 to identify key genes associated with the macrophage GSVA infiltration score. After correlation analysis, 2 lncRNAs and 21 mRNAs were found to have strong correlations with macrophage infiltration (Figure 7A). We then constructed a macrophage related ceRNA subnetwork by matching the miRNA targets of the 2 lncRNAs and the 23 mRNA which led to 29 lncRNA-miRNA-mRNA pairs including 2 lncRNAs, 14 miRNAs, and 18 mRNAs (Figure 7B).

### Prognosis analysis of macrophage related ceRNA subnetwork

**Figure 8:**
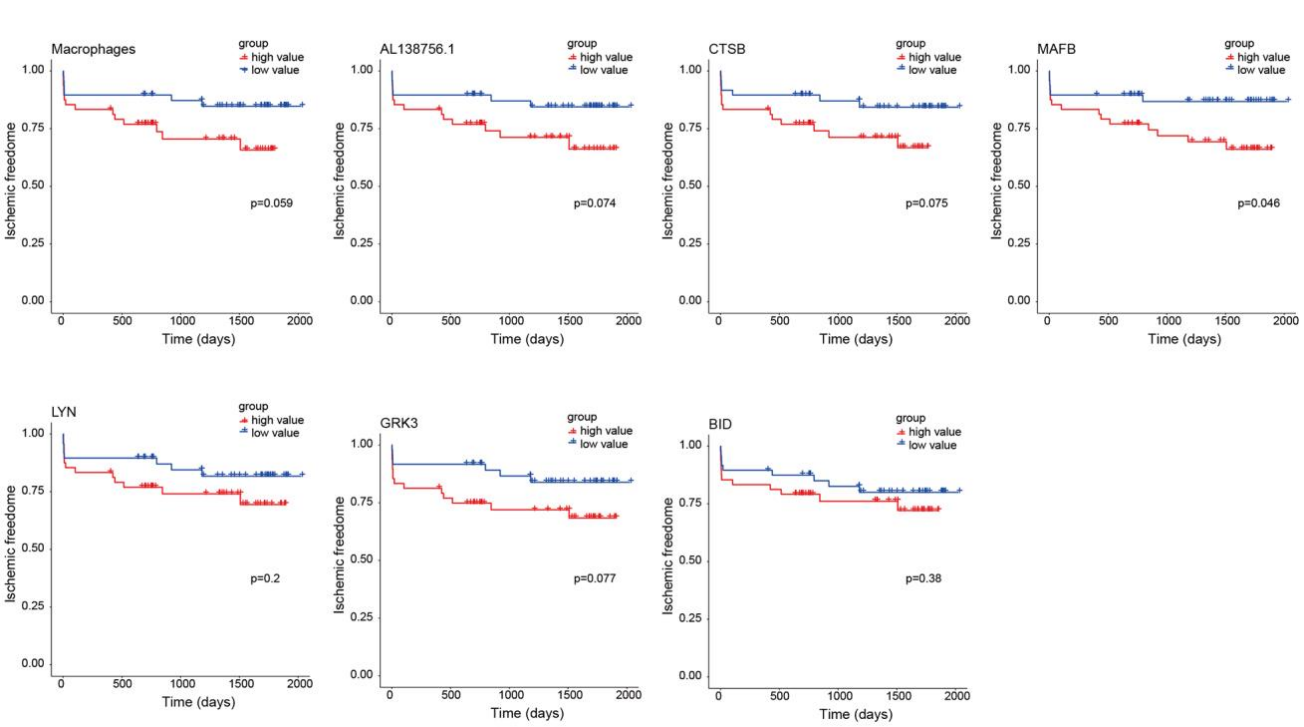
**Kaplan-Meier survival curves of 7 factors in the macrophage related ceRNA subnetwork.** The P-value was calculated by the log-rank test.

**Figure 9:**
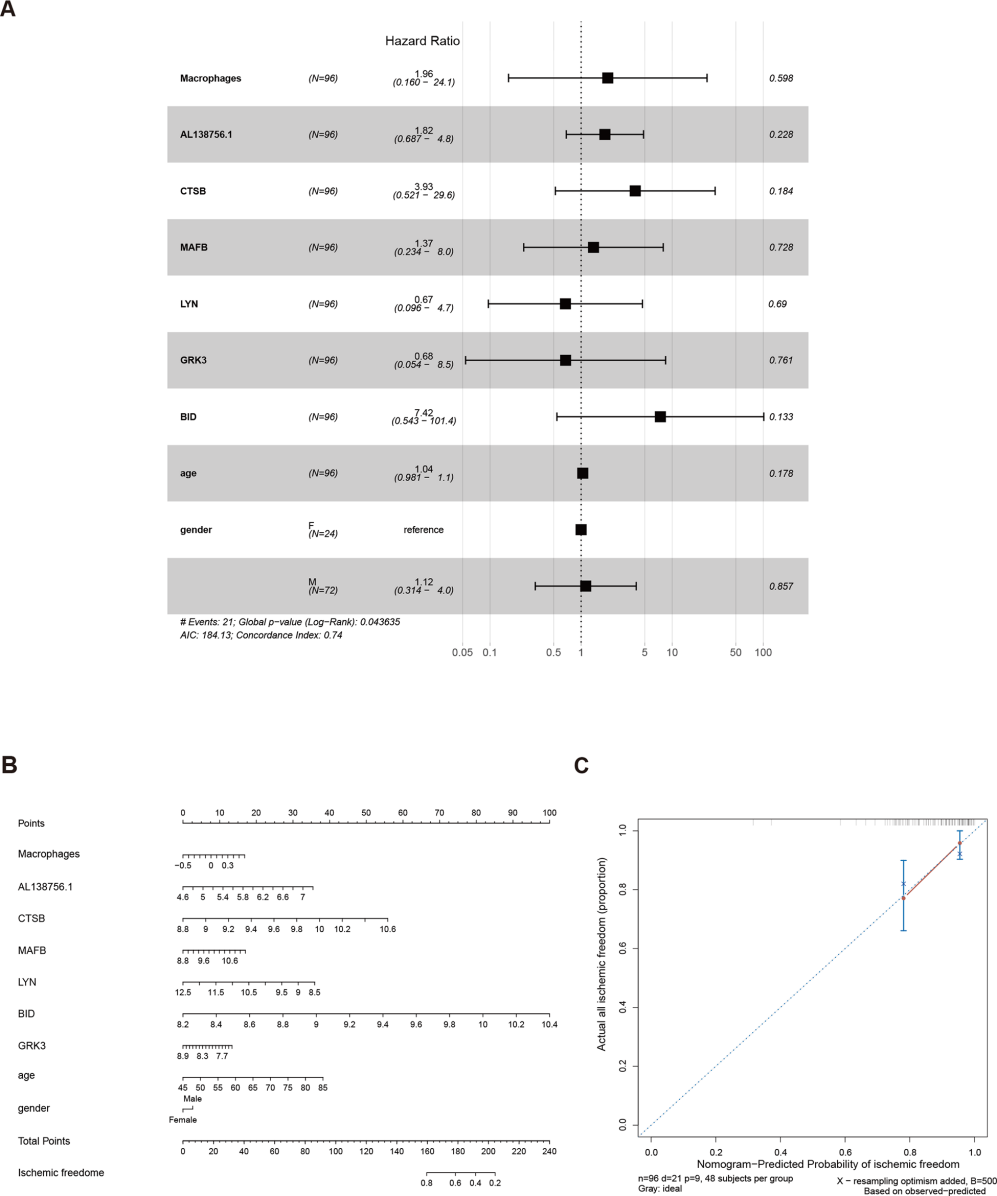
The results of the multivariate Cox regression (**A**), nomogram (**B**), and model diagnosis process of the calibration curve (**C**) constructed by the prognostic factors of the macrophage related ceRNA subnetwork (adjusted by age and gender).

As the constructed macrophage related ceRNA subnetwork may drive atherosclerosis plaque development and rupture, we then investigated their prognostic value to ischemic events. Univariate Cox regression analysis was used to identify the correlation between factors in the subnetwork and ischemic events. Through univariate Cox analysis, CTSB, macrophage GSVA score, AL138756.1, MAFB, LYN, GRK3, and BID were identified as crucial prognostic factors (Table 2). The Kaplan-Meier plots grouped by the median value of each factor were displayed in Figure 8. We then integrated them into a multivariate Cox regression model with age and gender-adjusted (Figure 9A). The C-index was tested as 0.74 which indicates the model to be reliable. Based on the model, a nomogram was established to predict the ischemic freedom probability of atherosclerosis patients (Figure 9B). The calibration curves (Figures 9C) were applied and manifested an acceptable calibration of the nomogram.

**Table 2:** Univariate cox regression analysis of members in macrophage related ceRNA subnetwork.

**Factor**		**HR**	**Lower (0.95)**	**Upper (0.95)**	**p**
CTSB		7.677	1.805	32.645	0.006 *
Macrophages		15.043	2.112	107.116	0.007 *
AL138756.1		2.899	1.182	7.109	0.020 *
MAFB		4.245	1.14	15.809	0.031 *
LYN		2.866	1.087	7.551	0.033 *
GRK3		7.556	1.14	50.089	0.036 *
BID		3.576	1.022	12.513	0.046 *
P2RX7		3.319	0.9	12.235	0.072
ADGRE2		1.88	0.913	3.872	0.087
CXCL16		1.763	0.843	3.688	0.132
LINC01094		1.615	0.841	3.103	0.150
LILRB2		1.884	0.721	4.921	0.196
CTSS		2.252	0.53	9.57	0.271
PYCARD		2.226	0.518	9.57	0.282
PNKD		1.534	0.592	3.976	0.379
BCAT1		1.475	0.59	3.689	0.406
CLEC7A		1.405	0.608	3.249	0.426
PSAP		1.706	0.345	8.443	0.513
SUSD1		1.411	0.319	6.235	0.65
GM2A		0.955	0.373	2.448	0.924
SRGAP2		0.985	0.196	4.953	0.985

*. p < 0.05.

### GSEA analysis

**Figure 10:**
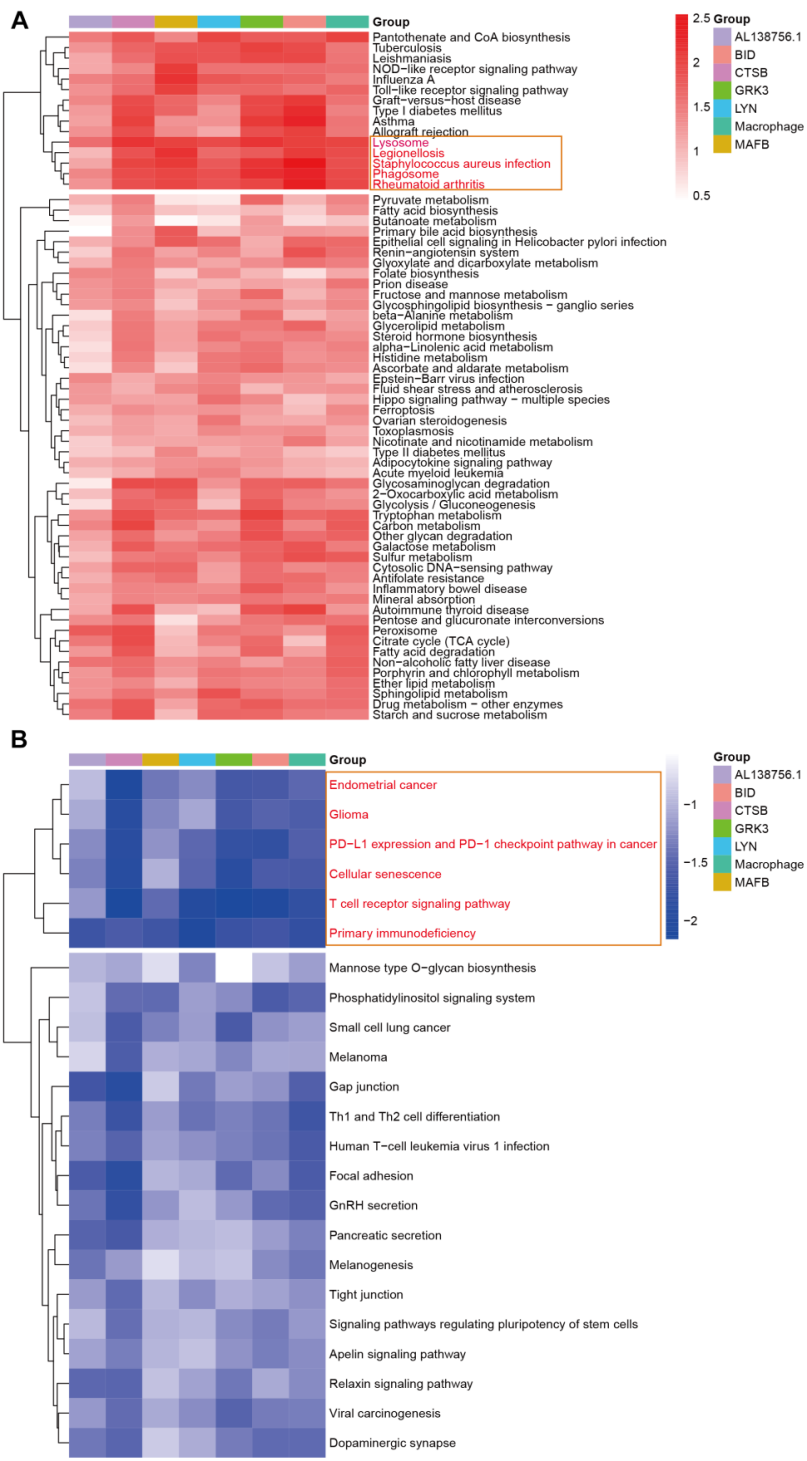
**The GSEA in response to AL138756.1, CTSB, MAFB, LYN, GRK3, BID, and macrophage GSVA score.** (**A**) shows the significant terms correlated with higher value, while (**B**) presents the opposite case.

**Figure 11:**
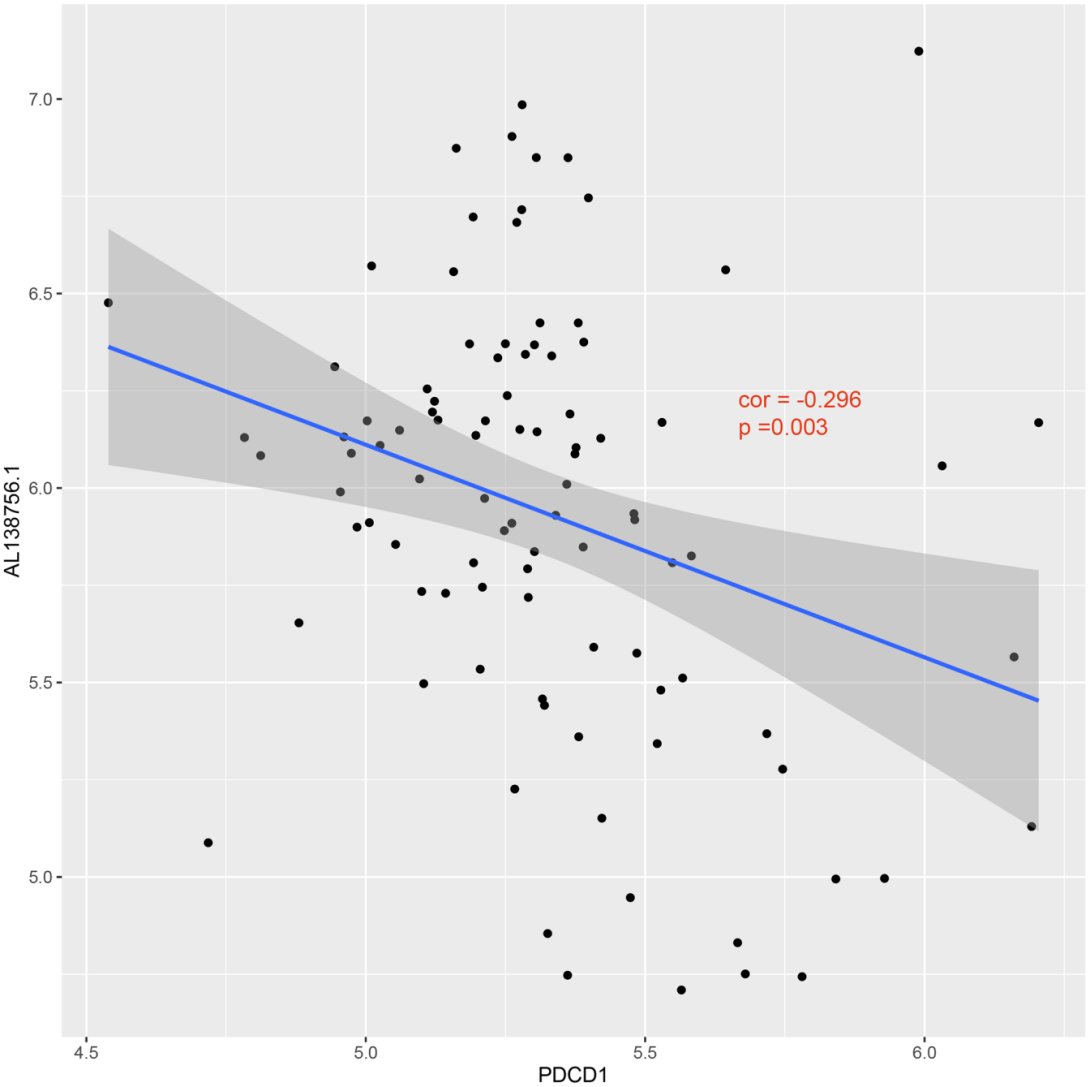
**Correlation between expression of PDCD1 and AL138756.1.**

The GSEA analysis was applied in GSE21545 to elucidate the regulatory pathways of the key prognostic factors after grouping by the median value of AL138756.1, CTSB, MAFB, LYN, GRK3, BID, and macrophage GSVA score. Through cluster analysis of the pathway NES values, phagosome and lysosome were significantly positively correlated with the expression value of AL138756.1, CTSB, MAFB, LYN, GRK3, BID, and macrophage GSVA score (Figure 10A). In contrast, the PD1 pathway, primary immunodeficiency were significantly negatively associated with these factors (Figure 10B). The negative correlation between PD1 signaling and the prognostic factors indicates that targeting the PD1 immune checkpoint during cancer immunotherapy may contribute to atherosclerosis development and ischemic risk through the macrophage related ceRNA subnetwork. Figure 11 further proves that a low level of PD1 might contribute to higher AL138756.1 expression.

## DISCUSSION

Atherosclerosis is initiated by the subendothelial retention of LDL, which then triggers a maladaptive inflammatory process that drives disease progression [16]. It is now understood that macrophage plays important roles in all stages of atherosclerosis, from initiation of lesions, to necrosis that leads to plaque rupture and the clinical manifestations, to resolution and regression of lesions [17]. In the current study, we identified a ceRNA immunoregulatory network in AS plaque development. We then established a macrophage related ceRNA subnetwork and identified several key factors that can be a prognostic tool to predict ischemic events including AL138756.1, CTSB, MAFB, LYN, GRK3, BID, and macrophage GSVA score.

To our knowledge, this is the first study to construct the ceRNA network in AS by using the method of WGCNA, the first to link ceRNA with immunoregulation, and the first to identify prognostic factors based on a ceRNA-immunoregulatory network. A previous study has used the differentially expressed gene (DEG) analysis to discover the ceRNA network in AS rabbits [18]. However, this method can be highly affected by the human-made criteria as a P-value and fold change must be set in advance, which ignores the high relationship between genes and may filter out those genes that have high interconnectivity in the network [19]. Our present study applied the WGCNA method and acquired the key modules of AS plaque development in which the lncRNAs and mRNAs were highly co-expressed. Therefore, it is more rational to construct the ceRNA network base on significant genes from the same module than simply using the results of DEG analysis.

Through enrichment analysis, the ceRNA network was mostly involved in the immune response. Therefore, we then conducted an immune infiltration analysis and identified macrophage as hub immunocyte in plaque development. In the established macrophage related ceRNA subnetwork, the two lncRNA AL138756.1 and LINC01094 lack sufficient investigation, while for the downregulated miRNAs, some of them have been implicated to be protective against AS. miR-106b can inhibit ox-LDL-induced endothelial cell apoptosis in AS [20]. It also inhibits the expression of PTEN in vascular ECs, which could block TNF-α-induced activation of caspase-3, thus preventing ECs apoptosis in AS [21]. miR-195-5p expressed in human aortic SMCs has the potential to inhibit SMC proliferation and migration in AS [22]. Thrombospondin 1 activates macrophage differentiation and SMC apoptosis [23] in human aortic dissection while decreases miR-25-5p expression in vascular SMCs [24]. miR-490-3p overexpression suppresses cell proliferation and inhibits proliferation and migration of vascular SMCs in AS [25]. ox-LDL inhibits the expression of miR-490-3p, resulting in vascular SMC proliferation on AS development [26].

Most of the mRNAs in the macrophage related ceRNA subnetwork have been reported in AS or macrophage related study. ADGRE2 (also known as EMR2), encodes for adhesion G protein-coupled receptor E2 and is expressed predominantly in macrophage [27]. EMR2 expression is upregulated during the differentiation and maturation of macrophage [28] and high levels of EMR2 have been detected in foamy macrophage in AS vessels [29]. A recent study indicated that macrophage BCAT1 (branched-chain amino acid transaminase 1) can interfere with metabolic reprogramming and was an attractive pharmacological target for the treatment of chronic inflammatory diseases [30]. CLEC7A (also known as Dectin-1), is a pattern recognition receptor necessary for the TLR2-mediated inflammatory response and enhances cytokine production in macrophage. Dectin-1 has been implicated to contribute to the oxidation of lipids and cholesterol accumulation in AS [31]. CTSS (cathepsin S) encodes a cysteine protease and is associated with lesion size, plaque vulnerability, and inflammatory markers in AS [32]. Of note, miR-106b-5p, the upstream of CTSS in the network, has been validated to bind to the 3'-untranslated region of CTSS while miR-106b-5p gain-of-function experiments lead to a decreased CTSS expression [33]. CXCL16 (C-X-C motif chemokine 16) significantly contributes to AS progression and functions as a biomarker for plaques at risk of rupture [34]. PYCARD, known as apoptosis-associated speck-like protein containing a CARD (ASC), is a component of NLRP3 inflammasome and is significantly increased in AS plaques compared to normal arteries [35]. Moreover, as the mRNAs are highly correlated, one could speculate that the upstream miRNA of one mRNA may affect other mRNAs. For instance, the miR-146a-3p targets BCAT1, GRK2 (G-protein-coupled receptor kinase 2), and P2RX7 (purinergic ligand-gated ion channel 7 receptor) in the network. These 3 mRNAs can therefore communicate with each other through ceRNA language. Studies have also proved that the miR-146a family can negatively regulate LPS-induced CXCL16 expression therefore preventing inflammation [36]. Interestingly, CXCL16 contributes to foam cell formation in the radial arteries and this process may be regulated by P2X7R [37]. These further support the high interconnections of the established ceRNA network and vouch for their importance in preventing macrophage-mediated AS development.

Through univariate Cox regression analysis, the lncRNA AL138756.1 and 5 mRNAs (CTSB, MAFB, LYN, GRK3, BID) were identified as prognostic biomarkers to ischemic events, which highlighted their importance in AS plaque development and rupture. Among them, the lncRNA AL138756.1 can upregulate the expression of CTSB, MAFB, LYN, and BID by competitively binding to miRNAs miR-1271-5p, miR-24-3p, let-7d-5p, and miR-195-3p/miR-507, respectively. Consistent with our results, previous studies suggested that CTSB (cathepsin B) contributed to AS vascular remodeling and plaque rupture [38, 39]. CTSB can regulate the activation of NLRP3 inflammasome [40, 41] and mediate macrophage promoted vascular inflammation and arterial remodeling. The CLARICOR study found that serum CTSB was associated with an increased risk of cardiovascular events in patients with stable coronary heart disease [42] and higher cathepsin activity along with an M2 macrophage phenotype was observed in carotid plaques from symptomatic patients [40]. MAFB is a transcription factor that regulates macrophage differentiation and function. It promotes AS by inhibiting foam-cell apoptosis in early plaque [43]. In advanced plaque, however, MAFB seems to be protective as macrophage-specific deletion of MAFB and exacerbated macrophage apoptosis in advanced AS results in more unstable and more vulnerable lesions because of defective efferocytosis [44]. Nevertheless, MAFB also seems to be related to atherogenesis through a macrophage-independent mechanism by exacerbating insulin resistance and lipid levels [45]. LYN (tyrosine-protein kinase Lyn) plays important role in CD36 mediated ox-LDL uptake by foam cell [46] and promotes AS. GRK3 belongs to the G protein-coupled receptor kinase family which serves broadly to regulate receptor function. BID is a member of the BCL-2 family of cell death regulators and promotes apoptosis. The functions of GRK3 and BID in atherosclerosis remain poorly investigated. The present study has pointed out their diagnostic and prognostic values.

We also conducted the GSEA analysis to identify key regulatory pathways of these prognostic factors. The results indicated that these factors were positively associated with phagocyte and lysome while negatively associated with PD1 signaling. Programmed cell death protein 1 (PD1, also known as PDCD1) is expressed during T cell activation and strongly interferes with T cell receptor signal transduction, thereby balancing protective immunity and immunopathology [47]. In animal studies, the PD-1 immune checkpoint has been implicated in protecting tissue tolerance while disruption of PD-1 associated negative signaling is associated with inflammatory disease including AS [48]. We found that the expression of AL138756.1 was significantly high when the PD1 immune checkpoint was down expressed. This may explain a recently published study in which targeting PD-1 signaling using monoclonal antibodies during cancer therapy leads to serious arterial inflammation [49].

There are limitations in our present study. Firstly, we didn’t conduct any validation and mechanism experiments, whether the identified association is a cause or result remains unclear. Besides, the miRNA profiles were retrieved from the platelets of patients with CAD. This is because no miRNAs feature of atherosclerotic plaque has been provided in GEO database. What’s more, the nomogram established has not been validated due to lacking external datasets. Further study can investigate their biological interaction in an experimental model and verify their prognostic values through a larger cohort.

In conclusion, our study innovatively linked ceRNA with immunoregulation in AS plaque development. Increased macrophage infiltration and higher expression of AL138756.1, CTSB, MAFB, LYN, GRK3, BID are associated with increased risk of ischemic events. These results will help us to better understand the mechanisms of immunoregulation in AS from the ceRNA perspective and provide candidate therapeutic and prognostic targets.

## MATERIALS AND METHODS

### Data acquisition and preprocessing

Three datasets, GSE21545, GSE28829, and GSE28858, were obtained from the NCBI Gene Expression Omnibus (GEO) (http://www.ncbi.nlm.nih.gov/geo). The GSE21545 dataset contains 97 peripheral blood mononuclear cells (PBMC) gene expression profiles from the Biobank of Karolinska Endarterectomy (BiKE) [50]. The 97 patients with atherosclerosis were followed for 1,159 ± 631 days (average ± standard deviation) and the ischemic events were defined as myocardial infarctions or ischemic strokes. GSM892606 were excluded due to the lack of age information and the rest 96 samples were selected for the following analysis. The GSE28829 dataset includes 16 advanced and 13 early atherosclerotic plaque samples from human carotid from the Maastricht Pathology Tissue Collection [51]. The GSE28858 dataset contains platelet miRNAs profiles from 12 patients with premature coronary arterial disease (CAD) and 12 age- and sex-matched healthy controls.

Both GSE21545 and GSE28829 were based on the GPL570 [HG-U133_Plus_2] Affymetrix Human Genome U133 Plus 2.0 Array platform. We reannotated the probes of GPL570 as it improves accuracy and makes it possible to identify new transcripts. In brief, the probe sequences were downloaded from Affymetrix (https://www.affymetrix.com) and were remapped to the human genome (GRCh38 release 99 primary assembly) using the R package ‘Rsubread’ [52]. Then, the chromosomal positions of these probes were matched to the corresponding genome annotation database in Ensembl using the R package ‘GenomicRanges’ [53]. Probe sets that were mapped to >1 gene were removed to ensure the reliability of the reannotation. For each dataset, the raw CEL files were preprocessed using robust multi-array average algorithm for background correction, quantile normalization, and summarization with ‘affy’ package [54]. The mean expression values among all multiple probe IDs were selected to represent the corresponding gene symbol. After that, 19557 unique genes were retained, which included 15394 protein coding genes and 3479 lncRNA according to the biotypes identified by Ensembl.

### Construction of weighted gene co-expression network

The expression profiles of GSE28829 were used to construct a gene co-expression network by using the package ‘WGCNA’ implemented in R software [55]. The top 50% mRNAs with the highest variability and all the lncRNAs were selected for further analysis. Subsequently, the power parameter ranging from 1-20 was screened out using the ‘pickSoftThreshold’ function. A suitable soft threshold of 10 was selected as it met the degree of independence of 0.85 with the minimum power value. The Dynamic Tree Cut method was applied to generate modules with the following major parameters to avoid the generation of too many modules: deepSplit of 2 and minModuleSize of 30. The height cut-off was set as 0.25, modules were merged if their similarity was >0.75. Ultimately, these mRNAs and lncRNAs in a co-expression module are considered to be highly interconnected. For each module, we conducted a gene ontology biological process enrichment analysis and the most significantly enriched term was summarized in Table 1.

### Relationship between clinical information and modules

The correlation between modules and clinical information (advanced or early atherosclerosis plaque) was identified by Pearson correlation’s analysis. For each module, module eigengenes (MEs) referred to the first principal component of all gene expression levels in the module, and therefore, it was reasonable to consider that MEs represented all genes within a specific module. We identified the association between MEs and external clinical information. If P-value was < 0.05, it was considered to be a significant correlation. Gene significance (GS) was defined as the correlation between gene expression and plaque status.

### CeRNA network construction and visualization

The expression file of GSE28858 was used to identify downregulated miRNAs in CAD using limma [56] packages in R with a criterion of P < 0.05 and CAD/healthy ratio < 1. Then, the downregulated miRNAs and lncRNAs/mRNAs in the most relevant module with a Gene significance >0.3/0.5 were used to construct a ceRNA network. Briefly, the associated ceRNA network in AF was constructed following three stages. (a) Prediction of lncRNA-miRNA: The potential miRNAs binding with lncRNAs were predicted based on the highly conserved microRNA family files of the miRcode [57] database (http://www.mircode.org/), and the primates conservative score should be >50%; (b) Prediction of miRNA-mRNA: We predicted the mRNAs binding with miRNAs identified from the previous step based on the annotation files of miRTarBase [58], which is one of the most comprehensively annotated and experimentally validated miRNA–target interaction databases.; (c) Construction of lncRNA-miRNA-mRNA ceRNA network: Cytoscape 3.7.2 software [59] (http://cytoscape.org/) was used to construct and visualize the ceRNA network based upon lncRNA-miRNA and miRNA-mRNA pairs.

### Gene ontology annotation, pathway enrichment, and protein-protein interaction analysis

Gene Ontology functional annotation and Reactome pathway enrichment analysis were conducted using the ‘ClusterProfiler’ [60] and ‘ReactomePA’ [61] packages in R solftware. To find out the functional associations among the identified genes, we used the online Search Tool for the Retrieval of Interacting Genes (STRING database; http://string-db.org/) to construct a protein-protein interaction (PPI) network based on uniquely comprehensive coverage and predictive function of genome-wide data [62]. A reliability threshold of a combined score of > 0.4 was used to construct the PPI network and Cytoscape software was used to visualize and analyze the biological networks.

### Computing the immune infiltration pattern

Two most popular methods were applied to quantify the infiltration of immune cell populations. The deconvolution method CIBERSORT [63] (https://cibersort.stanford.edu/) estimates the proportion of each immune cell population within the sample admixture, and the perm parameter was set as 1000. The Gene Set Variation Analysis (GSVA) [64] method computes the relative abundance of each immune cell population in each sample. We obtained gene signatures identifying 12 immune cell populations (B cells, Th1 cells, Th2 cells, Th17 cells, Tregs, CD8+ T cells, Macrophage, Neutrophils, Mast cells, Dendritic cells, NK cells, and Eosinophils) from the supplemental material of Bindea et al. [65]. Using the 12 gene sets, we computed the GSVA scores of each sample. Comparisons between Advanced and Early groups were tested by the Wilcoxon test, and cell type with P-value < 0.05 was considered significant.

### Construction of macrophage related ceRNA subnetwork

The correlation coefficient linking the infiltrating value of macrophage and the expression value of the 9 lncRNAs and 90 mRNAs in the ceRNA network were analyzed using Pearson’s method in 96 atherosclerosis samples from GSE21545. The key lncRNAs and mRNAs strongly correlated with macrophage infiltration were selected with a cor > 0.3 and p < 0.05. The corresponding lncRNA-miRNA-mRNA network was constructed and visualized using the ‘ggplot2’ [66] packages in R software.

### Prognosis analysis of the macrophage related ceRNA subnetwork

The 96 PBMC samples from GSE21545 were used for prognosis analysis. The macrophage infiltration scores of the 96 samples were computed by the GSVA method as previously described. The univariate Cox regression analysis was adopted to identify the prognostic values of lncRNAs, mRNAs, and macrophage infiltration score. Kaplan-Meier was used to visualize each biomarker by grouping the patients into high expression and low expression by their median value. Factors with p<0.05 in the univariate Cox model were integrated into a multivariable Cox proportional hazards regression analysis with the adjustments of age and gender. Then, a nomogram, based on the multivariable models, was built to predict the ischemic freedom of atherosclerosis patients using ‘rms’ package. To estimate the accuracy and discrimination of the nomogram, calibration curves and C-index test were applied.

### Identification of regulatory pathways of prognostic factors

After grouping by the median expression value of the key mRNA, lncRNA, and macrophage infiltration score, we applied the gene set enrichment analysis (GSEA) to detect the downstream KEGG (Kyoto Encyclopedia of Genes and Genomes) pathways. The normalized enrichment score (NES) was obtained and was used to identify key downstream regulatory pathways.

### Data availability statement

GSE21545, GSE28829, and GSE28858 can be downloaded from the GEO database. The results of WGCNA and R codes used in the present study were provided as Supplementary Materials 1 and 2.

## Supplementary Material

Supplementary Material 1

Supplementary Material 2
