# Supplementary material for: Constructing a ceRNA-immunoregulatory network associated with the development and prognosis of human atherosclerosis through weighted gene co-expression network analysis
Source: Aging (Albany NY). 2021 Jan 17;13(2):3080–100. doi: 10.18632/aging.202486 (PMC7880393; doi:10.18632/aging.202486)
Supplement: Supplementary Material 2 [file aging-13-202486-s002.pdf]

Constructing a ceRNA-immunoregulatory network associated with the development and prognosis of human atherosclerosis through weighted gene co-expression network analysis

Yaozhong Liu

2020/11/8

1 Statement

This code is only used for personal learning and replicating the study results. Do not use it for any commercial activity. If you have any problems in replicating the results, contact **Dr.Yaozhong Liu**, Second Xiangya Hospital, Central South University, email: *yaozhongliu@csu.edu.cn*.

2 Probe reannotation of GPL570

Before reannotation, download the original probe sequence file of GPL570; download the latest genomic fasta file 'Homo__sapiens.GRCh38.dna.primary__assembly.fa; download the latest genomic annotation file 'Homo__sapiens.GRCh38.99.gtf'.

```
library(GenomicRanges)
library(Rsubread)
library(Rsamtools)
```

2.1 Read the probe sequence file

```
gset <- read.table('GPL570.txt',sep = '\t', header = TRUE)
gset$Probe.Set.Name=as.character(gset$Probe.Set.Name)
gset$Probe.Sequence=as.character(gset$Probe.Sequence)
all_recs=paste(apply(gset,1,function(x) paste0('>',x[1],'\n',x[2])),collapse = '\n')
temp <- tempfile()
write(all_recs, temp)
```

2.2 Construct a index

This process is time consuming

```
dir='~/Probe_annotation'
ref <- file.path(dir,'Homo_sapiens.GRCh38.dna.primary_assembly.fa')
buildindex(basename="reference_index",reference=ref)
reads <- temp
align(index="reference_index",readfile1=reads,
      output_file="alignResults.GPL570.BAM",phredOffset=64)
propmapped("alignResults.GPL570.BAM")
```

2.3 Read the gtf file and make GenomicRanges files

```
gtf <- rtracklayer::import('Homo_sapiens.GRCh38.99.gtf')
gtf_df <- as.data.frame(gtf)
my_gr <- with(gtf_df, GRanges(as.character(seqnames), IRanges(start, end), as.character(strand), ensembl
bamFile="alignResults.GPL570.BAM"
quickBamFlagSummary(bamFile)
bam <- scanBam(bamFile)
tmp=as.data.frame(do.call(cbind,lapply(bam[[1]], as.character)))
tmp=tmp[tmp$flag!=4,]
my_seq <- with(tmp, GRanges(as.character(rname),IRanges(as.numeric(as.character(pos))-25,as.numeric(as.
```

2.4 Blast the two GenomicRanges files

```
gr3 = intersect(my_seq,my_gr)
o = findOverlaps(my_seq,my_gr)
lo=cbind(as.data.frame(my_seq[queryHits(o)]),as.data.frame(my_gr[subjectHits(o)]))
gpl570=lo[c(6,12,13,14)]
gpl570=gpl570[!duplicated(gpl570),]
```

2.5 Delect Probe sets that were mapped to >1 gene

```
gpl570=gpl570[,-2]
gpl570=gpl570[!duplicated(gpl570),]
gpl570$probe_id=as.character(gpl570$probe_id)
a=table(unlist(gpl570$probe_id))==1
b=data.frame(probe_id=a,probe_id1=a)
c=b[b$probe_id=='TRUE',]
c$probe_id=row.names(c)
d=merge(gpl570,c,by='probe_id')
uniqueProbe=d[,c(1,2,3)]
## Write tabl
write.table(uniqueProbe,file = 'uniqueprobetogene.txt',sep = '\t',row.names = F)
```

3 Data preprocessing

Download the raw cell files of pbmc samples from GSE21545 into the repository '~/GSE21545'. Download the raw cell files of carotid plaque samples from GSE28829 into the repository '~/GSE28829'.

3.1 GSE21545

```
setwd('~/GSE21545')
library(affyPLM)
Data=ReadAffy()
Datarma=rma(Data)
uniqueProbe=read.table("~/uniqueprobetogene.txt",sep = '\t',header = T)
exprSet <- as.data.frame(exprs(Datarma))
exprSet$probe_id <- rownames(exprSet)
exprSet_symbol <- merge(uniqueProbe,exprSet, by = "probe_id")
exprSet_symbol$probe_id <- NULL
#using median value to merge probes
exprSet_symbol <- aggregate(x = exprSet_symbol[,c(-1,-2)],
                            by = list(exprSet_symbol$gene_symbol), FUN="median")
rownames(exprSet_symbol)=exprSet_symbol$Group.1
exprSet_symbol=exprSet_symbol[,-1]
#write table
write.table(exprSet_symbol,file="unique_exprs_median.txt",sep = '\t')
```

3.2 GSE28829

```
setwd("~/GSE28829")
Data=ReadAffy(cdfname ='HG-U133_Plus_2' )
Datarma=rma(Data)
uniqueProbe=read.table("~/uniqueprobetogene.txt",sep = '\t',header = T)
exprSet <- as.data.frame(exprs(Datarma))
exprSet$probe_id <- rownames(exprSet)
exprSet_symbol <- merge(uniqueProbe,exprSet, by = "probe_id")
exprSet_symbol$probe_id <- NULL
#using median value to merge probes
exprSet_symbol<- aggregate(x = exprSet_symbol[,c(-1,-2)],
                           by = list(exprSet_symbol$gene_symbol),FUN="median")
rownames(exprSet_symbol)=exprSet_symbol$Group.1
exprSet_symbol=exprSet_symbol[,-1]
#write table
write.table(exprSet_symbol,file="unique_exprs_median.txt",sep = '\t')

#write table with gene type
exprSet_symbol$gene_symbol=rownames(exprSet_symbol)
exprSet_symbol_anno=merge(exprSet_symbol,uniqueProbe,by='gene_symbol')
exprSet_symbol_anno$probe_id=NULL
exprSet_symbol_anno=exprSet_symbol_anno[!duplicated(exprSet_symbol_anno),]
row.names(exprSet_symbol_anno)=exprSet_symbol_anno$gene_symbol
exprSet_symbol_anno$gene_symbol=NULL
write.table(exprSet_symbol_anno,file="unique_anno_exprs_median.txt",sep = '\t')
```

4 WGCNA

4.1 Select the top 50% mRNAs and all lncRNAs

```
exprs=read.table("~/GSE28829/unique_anno_exprs_median.txt",sep = "\t",row.names=1,header=T)
protein=exprs[exprs$gene_biotype=='protein_coding',]
lncRNA=exprs[exprs$gene_biotype=='lncRNA',]
#calculate the variance of mRNAs
protein$gene_biotype=apply(protein[,-30],1,var)
protein=protein[order(protein[,30],decreasing = T),]
#keep the top 50% mRNAs and all lncRNAs
exprstop=rbind(protein[1:round(15394/2),],lncRNA)
exprstop$gene_biotype=NULL
```

4.2 Select the power parameter

```
library(WGCNA)
datExpr=t(exprstop)
datExpr=data.frame(datExpr)
gsg = goodSamplesGenes(datExpr)
if (!gsg$allOK) {
  datExpr = datExpr[gsg$goodSamples, gsg$goodGenes]
}
nGenes = ncol(datExpr)
nSamples = nrow(datExpr)
#select the best power parameter
powers = c(c(1:10), seq(from = 12, to=20, by=2))
sft = pickSoftThreshold(datExpr, powerVector = powers, verbose = 5,blockSize = 20000)
str(sft)
#plot scale independence
par(mfrow=c(1,2))
plot(
  sft$fitIndices[,1],
  -sign(sft$fitIndices[,3])*sft$fitIndices[,2],
  xlab="Soft Threshold (power)",
  ylab="Scale Free Topology Model Fit,signed R^2",type="n",
  main = paste("Scale independence")
)
text(
  sft$fitIndices[,1],
  -sign(sft$fitIndices[,3])*sft$fitIndices[,2],
  labels=powers,
  cex=0.85,
  col="red"
)
abline(h=0.85, col="red")
# plot mean connectivity
plot(sft$fitIndices[,1], sft$fitIndices[,5],
     xlab="Soft Threshold (power)",ylab="Mean Connectivity", type="n",
     main = paste("Mean connectivity"))
```

```r
text(sft$fitIndices[,1], sft$fitIndices[,5], labels=powers,
     cex=0.85, col="red")
sft$powerEstimate
dev.off()
```

4.3 Run WGCNA

```r
cor <- WGCNA::cor
net = blockwiseModules(
  datExpr,
  power = sft$powerEstimate,
  maxBlockSize = 20000,
  TOMType = "unsigned",
  minModuleSize = 30,
  reassignThreshold = 0,
  mergeCutHeight = 0.25,
  numericLabels = TRUE,
  pamRespectsDendro = FALSE,
  saveTOMs =FALSE,
  verbose = 3)
cor<-stats::cor

#visualization
unmergedColors = labels2colors(net$unmergedColors)
mergedColors   = labels2colors(net$colors)
plotDendroAndColors(
  net$dendrograms[[1]],
  cbind(unmergedColors[net$blockGenes[[1]]], mergedColors[net$blockGenes[[1]]]),
  c("Dynamic Tree Cut" , "Merged colors"),
  dendroLabels = FALSE,
  hang = 0.03,
  addGuide = TRUE,
  guideHang = 0.05)

moduleColors = labels2colors(net$colors)
MEs = net$MEs
MEs_col = MEs
library(stringr)
colnames(MEs_col) = paste0("ME", labels2colors(
  as.numeric(str_replace_all(colnames(MEs),"ME",""))))
MEs_col = orderMEs(MEs_col)

#plot eigengene adjacency heatmap
plotEigengeneNetworks(MEs_col, "Eigengene adjacency heatmap",
                      marDendro = c(3,3,2,4),
                      marHeatmap = c(3,4,2,2), plotDendrograms = F,
                      xLabelsAngle = 90)
```

4.4 Relationship between clinical information and modules

```
class=c(rep("AD",16),rep("EAR",13))
design <- model.matrix(~-1+factor(class))
traitData=as.data.frame(design)
colnames(traitData)=c('Advanced plaque','Early plaque')
row.names(traitData)=row.names(datExpr)
corType = "pearson"
corFnc = ifelse(corType=="pearson", cor, bicor)
maxPOutliers = ifelse(corType=="pearson",1,0.05)
robustY = ifelse(corType=="pearson",T,F)
nGenes = ncol(datExpr)
nSamples = nrow(datExpr)
MEs0 = moduleEigengenes(datExpr, moduleColors)$eigengenes
MEs = orderMEs(MEs0)
moduleTraitCor = cor(
  MEs,
  traitData,
  use = "p"
)
moduleTraitPvalue = corPvalueStudent(
  moduleTraitCor,
  nSamples
)
textMatrix =  paste(
  signif(moduleTraitCor, 2),
  "\n(",
  signif(moduleTraitPvalue, 1),
  ")",
  sep = ""
)
dim(textMatrix) = dim(moduleTraitCor)
#plot
labeledHeatmap(Matrix = moduleTraitCor, xLabels = colnames(traitData),
               yLabels = colnames(MEs),
               cex.lab = 0.6,
               ySymbols = colnames(MEs), colorLabels = FALSE,
               colors = blueWhiteRed(50),
               textMatrix = textMatrix, setStdMargins = FALSE,
               cex.text = 0.6, zlim = c(-1,1),
               xLabelsAngle = 0,
               textAdj = c(0.5, 0.5),
               xLabelsAdj = 0.5,
               font.lab.x = 2,
               main = paste("Module-trait relationships"))
dev.off()
```

4.5 Results of WGCNA

```
#calculate connectivity
ADJ1 = abs(cor(datExpr,use = "p"))^6
```

```
Alldegrees = intramodularConnectivity(ADJ1,moduleColors)
head(Alldegrees)


#calculate module member-ship
datkME = as.data.frame(cor(datExpr, MEs, use ="p"))
head(datkME)

#calculate gene significance
GS = as.data.frame(cor(traitData$`Advanced plaque`,datExpr,use = "p"))
GS=as.data.frame(t(GS))
colnames(GS)='GS'

#write table
connectivity=as.data.frame(Alldegrees);MM=as.data.frame(datkME);color=as.data.frame(moduleColors)
key=cbind(connectivity,MM,color,GS)
row.names(key)=row.names(exprstop)
key$type=exprs[row.names(key),30]
write.table(key,file = "key_50_100_median.txt",sep = '\t')
```

4.6 GS-MM plot

```
key=read.table("~/key_50_100_median.txt",sep = '\t',header = T)
verboseScatterplot(abs(key[key$moduleColors=='red','MEred']), abs(key[key$moduleColors=='red','GS']),
                   xlab = 'Module Membership in red module', ylab = 'Gene significance for Advanced pla
                   main = 'Module membership vs. gene significance\n',
                   cex.main = 1, cex.lab = 1.2, cex.axis = 1.2, col = 'red')
```

4.7 GO BP analysis for each module

```
library(clusterProfiler)
library(org.Hs.eg.db)
GO=key[key$moduleColors=='red'&key$type=='protein_coding',]
ego <- enrichGO(
  gene          = row.names(GO),
  keyType = "SYMBOL",
  OrgDb         = org.Hs.eg.db,
  ont           = "BP",
  pAdjustMethod = "BH",
  pvalueCutoff  = 1,
  qvalueCutoff  = 1,
  readable      = FALSE)
head(as.data.frame(ego))
```

5 Construction of ceRNA network in red module

5.1 Identify downregulated miRNAs from GSE28858

GSE28858 was based on Illumina Human v2 MicroRNA expression beadchip. One should first download the expression matrix file of GSE28858 and the probe annotation file of chip platform. You should also transform the old miRNA name (based on miRbase release 12) to the latest name (based on miRbase release 22).

```
GSE28858=read.table(’GSE28828.txt’,sep = ’\t’,header = T)
library(limma)
class=c(rep("CAD",12),rep("NON",12))
design <- model.matrix(~-1+factor(class))
colnames(design) <- c("CAD","NON")
contrast.matrix=makeContrasts(contrasts = "CAD-NON",levels = design)
fit <- lmFit(GSE28858,design)
fit1=contrasts.fit(fit,contrast.matrix)
fit2 <- eBayes(fit1)
alldiff=topTable(fit2,adjust=’fdr’,coef="CAD-NON",number=200000)
#down regualted miRNAs
down_mirna=alldiff[alldiff$P.Value<0.05&alldiff$logFC<0,]
downmirna=unique(row.names(down_mirna))
```

5.2 Select the lncRNAs with GS>0.3 and mRNAs with GS>0.5 in red module

```
key=read.table("~/key_50_100_median.txt",sep = ’\t’,header = T)
key=key[key$moduleColors==’red’,]
#get the ensembl id of each gene symbol
annotation=read.table(’~/annotation.txt’,sep=’\t’,header=T)
#this file include three coloumns ’gene_id’,’gene_name" and ’gene_biotype’.
#one can make it from the latest gtf files.
key$gene_name=row.names(key)
key=merge(key,annotation,by=’gene_name’)
lncRNA=key[with(key,type==’lncRNA’),]
keylnc=key[key$gene_biotype==’lncRNA’&(key$GS>0.3),]
keyprotein=key[key$gene_biotype==’protein_coding’&&(key$GS>0.5),]
```

5.3 Match the key lncRNAs with downregulated miRNAs

download the miRcode database file ‘mircode_highconsfamilies.txt’

```
database=read.table("~/mircode_highconsfamilies.txt",sep = ’\t’,header = T)
database$ID=substring(database$gene_id,1,15)
database=database[database$primates_cons_.>50,]
lnc=database[with(database,database$ID %in% as.character(lncRNA$gene_id)),]
lnc=lnc[,c(1,4,13)]
lnc=lnc[!duplicated(lnc),]
#The format of miRNAs in miRcode database can not be directly compared.
#The below codes transform them into the latest miRbase 22 miRNA name. One can also compare them manual
library(stringr)
p1=str_starts(lnc$microrna,’miR-’)
```

```
p2=str_starts(lnc$microrna,'let-')
mis=lnc$microrna %>%
  str_remove('miR-') %>%
  str_remove('let-');head(mis)
mis=str_split(mis,'/');head(mis)
ln=sapply(mis, length);head(ln)
ps=list()
for (i in 1:length(ln)) {
  if(p1[[i]]){
    ps[[i]]=paste0('hsa-miR-',mis[[i]])
  }else{
    ps[[i]]=paste0('hsa-let-',mis[[i]])
  }
}
lnc2=data.frame(gene_id=rep(lnc$gene_id,times=ln),
                ID=rep(lnc$ID,times=ln),
                microrna=unlist(ps))

library(dplyr)
lnc2=distinct(lnc2)
mirna=as.character((lnc2$microrna))


z=list()
a=strsplit(as.character(mirna), split = "-")
ln=sapply(a, length)
for (i in 1:length(a)) {
  b=a[[i]][3]
  b=gsub('a','',b)
  b=gsub('b','',b)
  b=gsub('c','',b)
  b=gsub('d','',b)
  b=gsub('e','',b)
  b=gsub('f','',b)
  b=gsub('g','',b)
  b=gsub('h','',b)
  b=gsub('i','',b)
  n1=nchar(a[[i]][3])
  n2=nchar(b)
  n3=n1-n2
  if(ln[i]==4){
    if(n3==0){
      name=paste(a[[i]][1],a[[i]][2],a[[i]][3],a[[i]][4],sep = '-')
      z[[i]]=name
    }else{
      name1=paste(a[[i]][1],a[[i]][2],paste(substr(a[[i]][3],1,n2), substr(a[[i]][3],n2+1,n2+1),sep='')
      name2=paste(a[[i]][1],a[[i]][2],paste(substr(a[[i]][3],1,n2), substr(a[[i]][3],n2+2,n2+2),sep='')
      name3=paste(a[[i]][1],a[[i]][2],paste(substr(a[[i]][3],1,n2), substr(a[[i]][3],n2+3,n2+3),sep='')
      name4=paste(a[[i]][1],a[[i]][2],paste(substr(a[[i]][3],1,n2), substr(a[[i]][3],n2+4,n2+4),sep='')
      name5=paste(a[[i]][1],a[[i]][2],paste(substr(a[[i]][3],1,n2), substr(a[[i]][3],n2+5,n2+5),sep='')
      name6=paste(a[[i]][1],a[[i]][2],paste(substr(a[[i]][3],1,n2), substr(a[[i]][3],n2+6,n2+6),sep='')
      name7=paste(a[[i]][1],a[[i]][2],paste(substr(a[[i]][3],1,n2), substr(a[[i]][3],n2+7,n2+7),sep='')
      name8=paste(a[[i]][1],a[[i]][2],paste(substr(a[[i]][3],1,n2), substr(a[[i]][3],n2+8,n2+8),sep='')
```

```
      name9=paste(a[[i]][1],a[[i]][2],paste(substr(a[[i]][3],1,n2), substr(a[[i]][3],n2+9,n2+9),sep='')
      d=c(name1,name2,name3,name4,name5,name6,name7,name8,name9)
      d=unique(d)
      d=d[1:n3]
      z[[i]]=d
    }
  }else{
    if(n3==0){
      name=paste(a[[i]][1],a[[i]][2],a[[i]][3],sep = '-')
      z[[i]]=name
    }else{
      name1=paste(a[[i]][1],a[[i]][2],paste(substr(a[[i]][3],1,n2), substr(a[[i]][3],n2+1,n2+1),sep='')
      name2=paste(a[[i]][1],a[[i]][2],paste(substr(a[[i]][3],1,n2), substr(a[[i]][3],n2+2,n2+2),sep='')
      name3=paste(a[[i]][1],a[[i]][2],paste(substr(a[[i]][3],1,n2), substr(a[[i]][3],n2+3,n2+3),sep='')
      name4=paste(a[[i]][1],a[[i]][2],paste(substr(a[[i]][3],1,n2), substr(a[[i]][3],n2+4,n2+4),sep='')
      name5=paste(a[[i]][1],a[[i]][2],paste(substr(a[[i]][3],1,n2), substr(a[[i]][3],n2+5,n2+5),sep='')
      name6=paste(a[[i]][1],a[[i]][2],paste(substr(a[[i]][3],1,n2), substr(a[[i]][3],n2+6,n2+6),sep='')
      name7=paste(a[[i]][1],a[[i]][2],paste(substr(a[[i]][3],1,n2), substr(a[[i]][3],n2+7,n2+7),sep='')
      name8=paste(a[[i]][1],a[[i]][2],paste(substr(a[[i]][3],1,n2), substr(a[[i]][3],n2+8,n2+8),sep='')
      name9=paste(a[[i]][1],a[[i]][2],paste(substr(a[[i]][3],1,n2), substr(a[[i]][3],n2+9,n2+9),sep='')
      d=c(name1,name2,name3,name4,name5,name6,name7,name8,name9)
      d=unique(d)
      d=d[1:n3]
      z[[i]]=d
    }
  }
}

ln=sapply(z, length)
lnc3=data.frame(gene_id=rep(lnc2$gene_id,times=ln),
                ID=rep(lnc2$ID,times=ln),
                microrna=unlist(z))

lnc3=distinct(lnc3)

#download the miRbase release 22 file
miRNA=read.table("miRNA_22.txt",sep = '\t',header = T)
miRNA$ID=as.character(miRNA$ID)
human=miRNA[str_starts(miRNA$ID,'hsa-'),]
p1=str_starts(human$ID,'hsa-mir')
p2=str_starts(human$ID,'hsa-let')
human$ID=gsub('r','R',human$ID)

a=strsplit(as.character(human$ID), split = "-")
ln=sapply(a, length)
name1=list()
name2=list()
for (i in 1:length(a)) {
  b=a[[i]][3]
  b=gsub('a','',b)
  b=gsub('b','',b)
  b=gsub('c','',b)
  b=gsub('d','',b)
```

```
  b=gsub('e','',b)
  b=gsub('f','',b)
  b=gsub('g','',b)
  b=gsub('h','',b)
  b=gsub('i','',b)
  n1=nchar(a[[i]][3])
  n2=nchar(b)
  n3=n1-n2
  if(ln[i]==4){
      name1[[i]]=paste(a[[i]][1],a[[i]][2],a[[i]][3],a[[i]][4],sep = '-')
      name2[[i]]=paste(a[[i]][1],a[[i]][2],a[[i]][3],sep = '-')
  }else{
      name1[[i]]=paste(a[[i]][1],a[[i]][2],a[[i]][3],sep = '-')
      name2[[i]]=paste(a[[i]][1],a[[i]][2],b,sep = '-')
  }
}
human$name1=name1
human$name2=name2

#compare lnc3 with miRbase 22
a=strsplit(as.character(lnc3$microrna), split = "-")
b=as.character(lnc3$microrna)
mir=list()
ln=sapply(a, length)
for (i in 1:length(a)) {
  if(ln[[i]]==4){
    mir[[i]]=b[[i]]
  }else{
      c=b[[i]]
      if(c%in%human$name1){
        sub1=as.character(human[human$name1==c,]$Mature1_ID)
        sub2=as.character(human[human$name1==c,]$Mature2_ID)
        mir[[i]]=c(sub1,sub2)
        mir[[i]]=unique(mir[[i]])
      }else{
        sub1=as.character(human[human$name2==c,]$Mature1_ID)
        sub2=as.character(human[human$name2==c,]$Mature2_ID)
        mir[[i]]=c(sub1,sub2)
        mir[[i]]=unique(mir[[i]])
      }
    }
  }

#The final format
ln=sapply(mir, length)
lnc4=data.frame(gene_id=rep(lnc3$gene_id,times=ln),
                ID=rep(lnc3$ID,times=ln),
                microrna=unlist(mir))

lnc4=na.omit(lnc4)
lnc4=distinct(lnc4)

#Match the predicted miRNAs with downregulated miRNAs
```

```
lnc5=lnc4[lnc4$microrna%in%downmirna,]
```

5.4 Predict the mRNA targest of miRNAs

download the latest file from miRTarBase database

```
miRTarbase=read.table("~/human_tarbase_2020.txt",sep = '\t',header = TRUE)
miRTarbase=miRTarbase[as.character(miRTarbase$miRNA) %in%as.character(lnc5$microrna),]
miRTarbase$Species..miRNA.=NULL
miRTarbase=distinct(miRTarbase)
```

5.5 Macth the predicted mRNAs with key mRNAs in the red module

```
miRTarbase=miRTarbase[,c(2,3)];colnames(miRTarbase)=c("miRNA","mRNA")
miRTarbase=miRTarbase[miRTarbase$mRNA%in%as.character(keyprotein$gene_name),]
```

5.6 Final ceRNA network

```
lnc_mi=lnc5[,c(2,3)]
colnames(lnc_mi)=c("lncRNA","miRNA")
lnc_mi_m=merge(miRTarbase,lnc_mi,by="miRNA")
lnc_mi_m=lnc_mi_m[,c(3,1,2)]
n=length(colnames(lncRNA))
trans=lncRNA[,c(1,n-1)]
colnames(trans)=c('SYMBOL','lncRNA')
lnc_mi_m=merge(trans,lnc_mi_m,by='lncRNA')
levels(as.factor(as.character(lnc_mi_m$SYMBOL)))
levels(as.factor(as.character(lnc_mi_m$miRNA)))
levels(as.factor(as.character(lnc_mi_m$mRNA)))
#write table
write.table(lnc_mi_m,file="ceRNA.xls",row.names = F,sep = "\t")
```

5.7 GO ontology and Reactome enrichment analysis

```
library(clusterProfiler)
library(org.Hs.eg.db)
#GO biological process
GOBP <- enrichGO(
  gene          = unique(as.character(lnc_mi_m$mRNA)),
  keyType = "SYMBOL",
  OrgDb         = org.Hs.eg.db,
  ont           = "BP",
  pAdjustMethod = "BH",
  pvalueCutoff  = 1,
  qvalueCutoff  = 1,
  readable      = FALSE)
```

```r
barplot(GOBP, showCategory = 20)

#GO cellular component
GOCC <- enrichGO(
  gene          = unique(as.character(lnc_mi_m$mRNA)),
  keyType = "SYMBOL",
  OrgDb         = org.Hs.eg.db,
  ont           = "CC",
  pAdjustMethod = "BH",
  pvalueCutoff  = 1,
  qvalueCutoff  = 1,
  readable      = FALSE)
barplot(GOCC, showCategory = 20)

#GO molecular function
GOMF <- enrichGO(
  gene          = unique(as.character(lnc_mi_m$mRNA)),
  keyType = "SYMBOL",
  OrgDb         = org.Hs.eg.db,
  ont           = "MF",
  pAdjustMethod = "BH",
  pvalueCutoff  = 1,
  qvalueCutoff  = 1,
  readable      = FALSE)
barplot(GOMF, showCategory = 20)

#Reactome pathway
library(org.Hs.eg.db);library(clusterProfiler);library(ReactomePA)
gene.df <- bitr(unique(as.character(lnc_mi_m$mRNA)),
                fromType = "SYMBOL", toType = c("ENTREZID"),OrgDb = org.Hs.eg.db)
REACTOME=ReactomePA::enrichPathway(
  gene      = as.character(gene.df$ENTREZID),
  organism  = 'human',
  pAdjustMethod     = "BH",
  pvalueCutoff      = 1,
  qvalueCutoff  = 1,
  universe, minGSSize = 10,
  maxGSSize = 500,
)
barplot(REACTOME,showCategory = 20)
```

6 Immunocyte subtype infiltration detection

6.1 Detecting feature immunocyte in GSE28829 using GSVA

```r
library(GSVAdata)
library(Biobase)
library(RColorBrewer)
library(GSVA)
library(ggcorrplot)
```

```
library(ggthemes)
exprs=read.table("~/GSE28829/unique_exprs_median.txt",sep = "\t",row.names=1,header=T)
#read the gmt file of 12 immune cells
gmt_file="~/immune.cell.gmt"
geneset <- getGmt(gmt_file)
immune=gsva(as.matrix(exprs),geneset,min.sz=0, max.sz=5000, verbose=TRUE)
immune=as.data.frame(immune)
diffExpLevel=immune
annotation_col =data.frame(class=c(rep('AD',16),rep('EAR',13)))
row.names(annotation_col)=colnames(exprs)
library(pheatmap)
pheatmap(diffExpLevel,
         cluster_rows = F,
         cluster_cols = F,
         annotation_col =annotation_col,
         treeheight_col=10,
         annotation_legend=T,
         show_rownames = T,
         show_colnames = F,
         border_color = NA,
         scale = "none",
         color =colorRampPalette(c('#0099B4', '#ffffff','#ED0000'))(100)
dev.off()

#WILCOX rank test
for (i in 1:12){
  account=data.frame(X=as.numeric(immune[i,]),A=as.factor(annotation_col$class))
  cox=wilcox.test(X~A,data=account)
  print(row.names(immune)[[i]])
  print(cox)
}
```

6.2 Detecting feature immunocyte in GSE28829 using CIBERSORT

Use the expression file of GSE28829 to conduct immune infiltration analysis in 'https://cibersort.stanford.edu/' website. The R code for CIBERSORT and plotting won't be provided in here due to potential conflict interest.

7 Macrophage ceRNA network

7.1 Calculate GSVA score of macrophage in GSE21545

```
library(GSVAdata)
library(Biobase)
library(RColorBrewer)
library(GSVA)
library(ggcorrplot)
library(ggthemes)
exprs=read.table("~/GSE21545/unique_exprs_median.txt",sep = '\t',header = T)
#read the gmt file of 12 immune cells
```

```
gmt_file="~/immune.cell.gmt"
geneset <- getGmt(gmt_file)
immune=gsva(as.matrix(exprs),geneset,min.sz=0, max.sz=5000, verbose=TRUE)
immune=as.data.frame(immune)
#select the macrophage
macrophage=immune['Macrophages',]

#read the ceRNA network
lnc_mi_m=read.table("~/ceRNA.xls",header = T)
lncRNA=unique(as.character(lnc_mi_m$SYMBOL))
mRNA=unique((as.character(lnc_mi_m$mRNA)))
#correlation analysis
Vali=rbind(exprs[c(a,b),],macrophage)
Vali=na.omit(Vali)
corr=round(cor(t(Vali),method = 'pearson'),4)
p.mat=round(cor_pmat(t(Vali),method='pearson'),4)
pd=as.data.frame(p.mat)
cord=as.data.frame(corr)
#identify key lncRNAs and mRNAs strongly correlated with macrophage infiltration
#with a cor>0.3 and p<0.05.
A=pd[pd$Macrophages<0.05,]
B=cord[cord$Macrophages>0.3,]
D=intersect(row.names(A),row.names(B))
cordata=cord[D,D]
pdata=pd[D,D]

#plot the correlation map
ggcorrplot(cordata,hc.order = TRUE,hc.method = "ward.D",outline.color = "white",ggtheme = theme_bw(),
           type = "full",colors = c("#00468bff","white","#ad002aff"),lab = TRUE,lab_size = 2,
           p.mat = pdata,insig = "blank",tl.cex=6,)
```

7.2 Visualize the macrophage ceRNA network

```
macrocerna=lnc_mi_m[(lnc_mi_m$SYMBOL%in%D)&(lnc_mi_m$mRNA%in%D),]
ceRNA=macrocerna[,c(2:4)]
ceRNA$miRNA=substring(ceRNA$miRNA,5)
names(ceRNA)=c("lncRNA","miRNA","mRNA")
ceRNA$freg=as.numeric(as.factor(ceRNA$lncRNA))
da=ceRNA
library(ggalluvial)
library(ggsci)
ead(da)
is_alluvia_form(da,weight ="freg")
df_lodes <- to_lodes_form(da,key ="x", value = "stratum", id = "alluvium",axes =1:3)
head(df_lodes,12)
is_lodes_form(df_lodes,key = "x",value = "stratum",id = "alluvium",weight ="freg")


color_Pal  = pal_lancet("lanonc")(9)
mycol3 <- colorRampPalette(color_Pal)(34)
p5<-ggplot(df_lodes,aes(x = x, stratum =stratum, alluvium = alluvium,
```

```
                        fill = stratum, label = stratum)) +
  scale_x_discrete(expand = c(0, 0)) +
  geom_flow(width = 0.45, knot.pos = 0.2) +
  geom_stratum(alpha = .9,color="grey20",width = 3/7) +
  geom_text(stat = "stratum", size =3,color="black") +
  scale_fill_manual(values = mycol3) +
  xlab("") + ylab("") +
  theme_bw() +
  theme(panel.grid =element_blank()) +
  theme(panel.border = element_blank()) +
  theme(axis.line = element_blank(),axis.ticks =element_blank(),axis.text.y =element_blank())+
  guides(fill = FALSE)

p5
```

7.3 Prognosis analysis of the macrophage related ceRNA subnetwork

Get the following up information of patients and their age and gender information and write them into a 'sample.txt' file.

```
exprs=read.table('~GSE21545/unique_exprs_median.txt',sep = '\t',header = T)
gene=na.omit(exprs[c(unique(as.character(macrocerna$mRNA)),unique(as.character(macrocerna$SYMBOL))),])
immune=cbind(gene,macrophage)
immune=as.data.frame(t(immune))
sample=read.table('~/sample.txt',sep = '\t',header = T)
prognosis=cbind(sample,immune)
```

7.3.1 univariate Cox analysis

```
beta_co=c()
HR=c()
p=c()
lower=c()
upper=c()
for (i in 1:length(colnames(immune))) {
  candidatelnc=colnames(immune)[[i]]
  formula=as.formula(paste0('Surv(days,outcome)~',candidatelnc))
  surv_uni_cox=summary(coxph(formula,data = prognosis))
  ph_hypothesis_p=cox.zph(coxph(formula,data = prognosis))$table[1,3]
  beta_co=append(beta_co,surv_uni_cox$coefficients[,1])
  HR=append(HR,exp(surv_uni_cox$coefficients[,1]))
  p=append(p,surv_uni_cox$coefficients[,5])
  lower=append(lower,surv_uni_cox$conf.int[,3])
  upper=append(upper,surv_uni_cox$conf.int[,4])          }

unicox=data.frame(HR=HR,lower=lower,upper=upper,p=p)
row.names(unicox)=colnames(immune)
unicox=round(unicox,3)
```

7.3.2 KM analysis

```
library(survival)
library('survminer')
setwd('~/KM')
for (i in 1:length(colnames(immune))) {
  candidate=colnames(immune)[[i]]
  high_low=(immune[,i]>median(immune[,i]))
  high_low[high_low==TRUE]='high'
  high_low[high_low==FALSE]='low'
  risk_score_table=cbind(sample[,c(5,7)],high_low)
  fit_km=survfit(Surv(days,outcome)~high_low,data = risk_score_table)
  pdf(file=paste('KM',candidatelnc,'.pdf'))
  A=ggsurvplot(fit_km,conf.int = F,pval = T,title=candidate,
               legend=c(0.8, 1),legend.labs=c('high value','low value'),
               risk.table = F,legend.title='group',
               palette = c('red','blue'),surv.median.line = 'none')
  print(A)
  dev.off()
}
```

7.3.3 multivariate Cox analysis adjusted by age and gender

```
formula=Surv(days,outcome)~Macrophages+AL138756.1+CTSB+MAFB+LYN+GRK3+BID+age+gender
multi_variate_cox=coxph(formula,data=prognosis)
ph_hypothesis_p=cox.zph(coxph(formula,data = pbmc))$table[1,3]
print(summary(multi_variate_cox)$coefficients)
print(ph_hypothesis_p)
#plot forest map
library(survminer)
ggforest(model=multi_variate_cox,data=prognosis,main='Hazard Ratio')
```

7.3.4 nomogram

```
library(Hmisc); library(grid); library(lattice);library(Formula); library(ggplot2)
library(rms)
library(survival)
nomo=prognosis
nomo$gender <- as.factor(ifelse(nomo$gender=='M',"Male","Female"))
dd<-datadist(nomo)
options(datadist='dd')
coxm <- cph(Surv(nomo$days,nomo$outcome)~Macrophages+AL138756.1+CTSB+MAFB+LYN+BID+GRK3+age+gender,
            x=T,y=T,data=nomo,surv = T)
surv <- Survival(coxm)
surv1 <- function(x)surv(2026,lp=x)
surv2 <- function(x)surv(5*365,lp=x)
plot(nomogram(coxm,fun=list(surv1),lp= F,funlabel=c('Ischemic freedome'),maxscale=100,fun.at=seq(0,1,0.[illegible]
```

7.3.5 calibration curves

```
cal =calibrate(coxm, u=250,cmethod = 'KM',method = 'boot' , m = 48, B = 500)
plot(cal,lwd=2,lty=1,
     errbar.col=c(rgb(0,118,192,maxColorValue=255)),
     xlim=c(0,1),ylim=c(0,1),
     xlab="Nomogram-Predicted Probability of ischemic freedom",
     ylab="Actual ischemic freedom (proportion)",
     col=c(rgb(192,98,83,10,maxColorValue=255)))
lines(cal[,c('mean.predicted','KM')],type = 'b',lwd = 2,col = c(rgb(192,98,83,maxColorValue = 255)),pch
abline(0,1,lty = 3,lwd = 2,col = c(rgb(0,118,192,maxColorValue = 255)))
#C index
validate(coxm, method="randomization", B=1000, dxy=T)
1-rcorrcens(Surv(days,outcome) ~ predict(coxm), data =  nomo)
```

7.4 GSEA analysis

```
exprs=read.table('~/GSE21545/unique_exprs_median.txt',sep = '\t',header = T)
exprs1=exprs[c('AL138756.1','CTSB','MAFB','LYN','GRK3','BID'),]
macrocerna=rbind(exprs1,macrophage)
```

7.4.1 group sample by median values

```
macrocerna=as.data.frame(t(macrocerna))
macrocerna$AL138756.1=as.factor(ifelse(macrocerna$AL138756.1>median(macrocerna$AL138756.1),'High','Low'
macrocerna$CTSB=as.factor(ifelse(macrocerna$CTSB>median(macrocerna$CTSB),'High','Low'))
macrocerna$MAFB=as.factor(ifelse(macrocerna$MAFB>median(macrocerna$MAFB),'High','Low'))
macrocerna$LYN=as.factor(ifelse(macrocerna$LYN>median(macrocerna$LYN),'High','Low'))
macrocerna$GRK3=as.factor(ifelse(macrocerna$GRK3>median(macrocerna$GRK3),'High','Low'))
macrocerna$BID=as.factor(ifelse(macrocerna$BID>median(macrocerna$BID),'High','Low'))
macrocerna$Macrophages=as.factor(ifelse(macrocerna$Macrophages>median(macrocerna$Macrophages),'High','L
```

7.4.2 get ranked gene list

```
library(limma);library(org.Hs.eg.db);library(clusterProfiler)
gene.df <- bitr(row.names(exprs), fromType = "SYMBOL",
                toType = c("ENTREZID"),
                OrgDb = org.Hs.eg.db)
# group by AL138756.1
design <- model.matrix(~-1+factor(macrocerna$AL138756.1))
colnames(design) <- c("High","Low")
contrast.matrix=makeContrasts(contrasts = "High-Low",levels = design)
fit <- lmFit(exprs,design)
fit1=contrasts.fit(fit,contrast.matrix)
fit2 <- eBayes(fit1)
allDiff=topTable(fit2,adjust='fdr',coef="High-Low",number=200000)
```

```
allDiff$SYMBOL=row.names(allDiff)
allDiff=merge(allDiff,gene.df,by='SYMBOL')
row.names(allDiff)=allDiff$ENTREZID
allDiff=allDiff[order(allDiff$logFC,decreasing = T),]
genelist1=as.vector(allDiff$logFC)
names(genelist1)=row.names(allDiff)

#group by CTSB
design <- model.matrix(~-1+factor(macrocerna$CTSB))
colnames(design) <- c("High","Low")
contrast.matrix=makeContrasts(contrasts = "High-Low",levels = design)
fit <- lmFit(exprs,design)
fit1=contrasts.fit(fit,contrast.matrix)
fit2 <- eBayes(fit1)
allDiff=topTable(fit2,adjust='fdr',coef="High-Low",number=200000)
allDiff$SYMBOL=row.names(allDiff)
allDiff=merge(allDiff,gene.df,by='SYMBOL')
row.names(allDiff)=allDiff$ENTREZID
allDiff=allDiff[order(allDiff$logFC,decreasing = T),]
genelist2=as.vector(allDiff$logFC)
names(genelist2)=row.names(allDiff)

#group by MAFB
design <- model.matrix(~-1+factor(macrocerna$MAFB))
colnames(design) <- c("High","Low")
contrast.matrix=makeContrasts(contrasts = "High-Low",levels = design)
fit <- lmFit(exprs,design)
fit1=contrasts.fit(fit,contrast.matrix)
fit2 <- eBayes(fit1)
allDiff=topTable(fit2,adjust='fdr',coef="High-Low",number=200000)
allDiff$SYMBOL=row.names(allDiff)
allDiff=merge(allDiff,gene.df,by='SYMBOL')
row.names(allDiff)=allDiff$ENTREZID
allDiff=allDiff[order(allDiff$logFC,decreasing = T),]
genelist3=as.vector(allDiff$logFC)
names(genelist3)=row.names(allDiff)

#group by LYN
design <- model.matrix(~-1+factor(macrocerna$LYN))
colnames(design) <- c("High","Low")
contrast.matrix=makeContrasts(contrasts = "High-Low",levels = design)
fit <- lmFit(exprs,design)
fit1=contrasts.fit(fit,contrast.matrix)
fit2 <- eBayes(fit1)
allDiff=topTable(fit2,adjust='fdr',coef="High-Low",number=200000)
allDiff$SYMBOL=row.names(allDiff)
allDiff=merge(allDiff,gene.df,by='SYMBOL')
row.names(allDiff)=allDiff$ENTREZID
allDiff=allDiff[order(allDiff$logFC,decreasing = T),]
genelist4=as.vector(allDiff$logFC)
names(genelist4)=row.names(allDiff)

#group by GRK3
```

```
design <- model.matrix(~-1+factor(macrocerna$GRK3))
colnames(design) <- c("High","Low")
contrast.matrix=makeContrasts(contrasts = "High-Low",levels = design)
fit <- lmFit(exprs,design)
fit1=contrasts.fit(fit,contrast.matrix)
fit2 <- eBayes(fit1)
allDiff=topTable(fit2,adjust='fdr',coef="High-Low",number=200000)
allDiff$SYMBOL=row.names(allDiff)
allDiff=merge(allDiff,gene.df,by='SYMBOL')
row.names(allDiff)=allDiff$ENTREZID
allDiff=allDiff[order(allDiff$logFC,decreasing = T),]
genelist5=as.vector(allDiff$logFC)
names(genelist5)=row.names(allDiff)

#group by BID
design <- model.matrix(~-1+factor(macrocerna$BID))
colnames(design) <- c("High","Low")
contrast.matrix=makeContrasts(contrasts = "High-Low",levels = design)
fit <- lmFit(exprs,design)
fit1=contrasts.fit(fit,contrast.matrix)
fit2 <- eBayes(fit1)
allDiff=topTable(fit2,adjust='fdr',coef="High-Low",number=200000)
allDiff$SYMBOL=row.names(allDiff)
allDiff=merge(allDiff,gene.df,by='SYMBOL')
row.names(allDiff)=allDiff$ENTREZID
allDiff=allDiff[order(allDiff$logFC,decreasing = T),]
genelist6=as.vector(allDiff$logFC)
names(genelist6)=row.names(allDiff)

#group by Macrophages
design <- model.matrix(~-1+factor(macrocerna$Macrophages))
colnames(design) <- c("High","Low")
contrast.matrix=makeContrasts(contrasts = "High-Low",levels = design)
fit <- lmFit(exprs,design)
fit1=contrasts.fit(fit,contrast.matrix)
fit2 <- eBayes(fit1)
allDiff=topTable(fit2,adjust='fdr',coef="High-Low",number=200000)
allDiff$SYMBOL=row.names(allDiff)
allDiff=merge(allDiff,gene.df,by='SYMBOL')
row.names(allDiff)=allDiff$ENTREZID
allDiff=allDiff[order(allDiff$logFC,decreasing = T),]
genelist7=as.vector(allDiff$logFC)
names(genelist7)=row.names(allDiff)
```

7.4.3 GSEA analysis

```
KEGG1=gseKEGG(genelist1,nPerm = 1000,minGSSize = 10,maxGSSize = 500,pvalueCutoff = 1,use_internal_data=
KEGG2=gseKEGG(genelist2,nPerm = 1000,minGSSize = 10,maxGSSize = 500,pvalueCutoff = 1,use_internal_data=
KEGG3=gseKEGG(genelist3,nPerm = 1000,minGSSize = 10,maxGSSize = 500,pvalueCutoff = 1,use_internal_data=
KEGG4=gseKEGG(genelist4,nPerm = 1000,minGSSize = 10,maxGSSize = 500,pvalueCutoff = 1,use_internal_data=
KEGG5=gseKEGG(genelist5,nPerm = 1000,minGSSize = 10,maxGSSize = 500,pvalueCutoff = 1,use_internal_data=
```

```
KEGG6=gseKEGG(genelist6,nPerm = 1000,minGSSize = 10,maxGSSize = 500,pvalueCutoff = 1,use_internal_data=
KEGG7=gseKEGG(genelist7,nPerm = 1000,minGSSize = 10,maxGSSize = 500,pvalueCutoff = 1,use_internal_data=

a1=as.data.frame(KEGG1);a1=a1[a1$pvalue<1,c(2,5)];row.names(a1)=a1$Description
a2=as.data.frame(KEGG2);a2=a2[a2$pvalue<1,c(2,5)];row.names(a2)=a2$Description
a3=as.data.frame(KEGG3);a3=a3[a3$pvalue<1,c(2,5)];row.names(a3)=a3$Description
a4=as.data.frame(KEGG4);a4=a4[a4$pvalue<1,c(2,5)];row.names(a4)=a4$Description
a5=as.data.frame(KEGG5);a5=a5[a5$pvalue<1,c(2,5)];row.names(a5)=a5$Description
a6=as.data.frame(KEGG6);a6=a6[a6$pvalue<1,c(2,5)];row.names(a6)=a6$Description
a7=as.data.frame(KEGG7);a7=a7[a7$pvalue<1,c(2,5)];row.names(a7)=a7$Description

a8=merge(a1,a2,by='Description')
a9=merge(a8,a3,by='Description')
a10=merge(a9,a4,by='Description')
a11=merge(a10,a5,by='Description')
a12=merge(a11,a6,by='Description')
a13=merge(a12,a7,by='Description')

row.names(a13)=a13$Description
a13$Description=NULL
colnames(a13)=c('AL138756.1','CTSB','MAFB','LYN','GRK3','BID','Macrophage')
annotation_col <-data.frame(Group=c('AL138756.1','CTSB','MAFB','LYN','GRK3','BID','Macrophage'))
rownames(annotation_col) <- colnames(a13)

a14=a13[a13$AL138756.1>0&a13$CTSB>0&a13$MAFB>0&a13$LYN>0&a13$GRK3>0&a13$BID>0&a13$Macrophage>0,]
a15=a13[a13$AL138756.1<0&a13$CTSB<0&a13$MAFB<0&a13$LYN<0&a13$GRK3<0&a13$BID<0&a13$Macrophage<0,]

#plot positively related pathway
diffExpLevel=a14[order(apply(a14,1,sum),decreasing = T),]
library(pheatmap)
pheatmap(diffExpLevel,
         cluster_rows = T,
         cluster_cols = F,
         annotation_col =annotation_col,
         annotation_legend=T,
         show_rownames = T,
         show_colnames = F,
         border_color = NA,
         scale = "none",
         cutree_rows=2,
         treeheight_col=10,
         color =colorRampPalette(c("white",'red'))(100)
         )

#plot negatively related pathway
diffExpLevel=a15[order(apply(a15,1,sum),decreasing = F),]
library(pheatmap)
pheatmap(diffExpLevel,
         cluster_rows = T,
         cluster_cols = F,
         annotation_col =annotation_col,
         annotation_legend=T,
         show_rownames = T,
```

```
         show_colnames = F,
         border_color = NA,
         scale = "none",
         cutree_rows=2,
         treeheight_col=10,
         color =colorRampPalette(c("blue",'white'))(100)
         )
```

7.4.4 identify the correlation between AL138756.1 and PDCD1

```
macrocerna=rbind(exprs1,immune)
PD1=data.frame(PDCD1=as.numeric(exprs['PDCD1',]),AL138756.1=as.numeric(macrocerna['AL138756.1',]))
ggplot(PD1,aes(x=PDCD1,y=AL138756.1))+geom_point()+geom_smooth(method =lm)
```

8 Acknowledgement

Yaozhong Liu would like to thank Ziwei Wan for her love and thoughtful kindness.
